# Changes in the lipid profile of hamster liver after *Schistosoma mansoni* infection, characterized by mass spectrometry imaging and LC–MS/MS analysis

**DOI:** 10.1007/s00216-022-04006-6

**Published:** 2022-03-23

**Authors:** Katja R. Wiedemann, Alejandra Peter Ventura, Stefanie Gerbig, Martin Roderfeld, Thomas Quack, Christoph G. Grevelding, Elke Roeb, Bernhard Spengler

**Affiliations:** 1 Institute of Inorganic and Analytical Chemistry, Justus Liebig University Giessen, Giessen, Germany; 2 Gastroenterology, Justus Liebig University Giessen, Giessen, Germany; 3 Institute for Parasitology, Justus Liebig University Giessen, Giessen, Germany

**Keywords:** Schistosomiasis, AP-SMALDI, Mass spectrometry imaging, Infection, Host-parasite interaction, Granuloma, Parasites, *Schistosoma mansoni*

## Abstract

Schistosomiasis, caused by the human parasite *Schistosoma mansoni*, is one of the WHO-listed neglected tropical diseases (NTDs), and it has severe impact on morbidity and mortality, especially in Africa. Not only the adult worms but also their eggs are responsible for health problems. Up to 50% of the eggs produced by the female worms are not excreted with the feces but are trapped in the host tissue, such as the liver, where they provoke immune responses and a change in the lipid profile. We built up a database with 372 infection markers found in livers of *S. mansoni*-infected hamsters, using LC–MS/MS for identification, followed by statistical analysis. Most of them belong to the lipid classes of phosphatidylcholines (PCs), phosphatidylethanolamines (PEs), and triglycerides (TGs). We assigned some of these markers to specific anatomical structures by applying high-resolution MALDI MSI to cryosections of hamster liver and generating ion images based on the marker list from the LC–MS/MS experiments. Furthermore, enrichment and depletion of several markers were visualized.

**Graphic Abstract:**

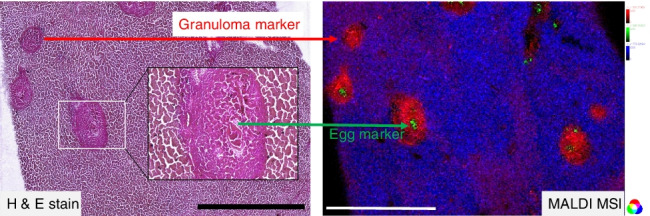

**Supplementary Information:**

The online version contains supplementary material available at 10.1007/s00216-022-04006-6.

## Introduction

Infection with the blood fluke *Schistosoma mansoni* leads to severe health issues. The resulting disease schistosomiasis can occur in two different stages, an acute and a chronic form. During acute infection, patients typically suffer from non-specific symptoms such as fever, fatigue, malaise, and cough. When the worms start to migrate, abdominal symptoms can occur [1]. Main symptoms of chronic infection are abdominal pain, diarrhea or bloody stool, and inflammation of inner organs such as the liver, spleen, and gut [2].

Humans infected with *S.* *mansoni* excrete parasite eggs with their feces. When the eggs enter fresh water, the first larval stage, miracidia hatch and infect their intermediate host, a snail of the genus *Biomphalaria*. Inside the snails, the parasite multiplies asexually and develops after 4 to 6 weeks to male and female cercariae, which are then released into water. Cercariae penetrate the skin of their vertebrate host and migrate to the blood vessels. During this phase, cercariae transform into schistosomula and subsequently to adult schistosomes. In the portal vein of the liver, male and female worms mate and migrate as couples to the mesenteric veins of the gut, where they can live for up to 30 years. A unique feature of schistosome biology is that the sexual maturation of the female is only achieved upon a constant pairing contact with the male. Following pairing, the female reproductive organs fully differentiate; the female starts the production of several hundred eggs per day, which reach the gut lumen and are excreted by the host [1, 2].

Schistosomiasis is classified as an endemic disease being prevalent in tropical and subtropical regions worldwide. According to the WHO, schistosomiasis belongs to the NTDs and is mainly spread in Africa, Asia, the Middle East, the Caribbean, and parts of South America [2, 3].

Improving both hygienic conditions as well as developing chemotherapeutics plays an important role in combating schistosomiasis and preventing the further spread of the disease [1]. Since 1977, praziquantel (PZQ) is the main drug against schistosomiasis [4]. PZQ is effectively targeting all schistosome species, but due to the risk of upcoming resistances against PZQ, new drugs are urgently needed. Additionally, PZQ affects adult but not juvenile worms [5].

In case of *S. mansoni*, about 50% of the eggs are excreted with the feces, the other 50% are mostly trapped in the liver and intestine of the host and secrete antigens and other factors, which can affect the host [6, 7]. Trapped eggs are the primary reason for the pathological consequences of schistosomiasis. While the host’s immune response is mainly directed towards antigens produced by the schistosome worms during the first weeks of infection, the immune response shifts after 5 to 6 weeks. After pairing, female schistosomes start to produce eggs, which then provoke the type 2 response of the host’s immune system [7, 8]. This immune response is essential for the egg’s movement through the intestinal wall to reach the gut lumen [7]. Due to the reaction of the tissue, granuloma formation occurs around the eggs within a few days [1]. Granuloma have a dual function as they appear to be important for the survival of the host by protecting hepatocytes from toxins released by the eggs. In addition, granuloma results from inflammatory processes that finally lead to liver fibrosis [7]. Granuloma in general mainly consists of macrophages which mass together [9]. However, liver granulomas are more heterogenic in cell types and contain also T and B lymphocytes as well as eosinophils and mast cells [10]. Furthermore, it appears that granuloma formation differs in naturally infected hosts from those observed under laboratory conditions [11]. It is known that *S.* *mansoni* infection in general leads to lower cholesterol, low-density lipoprotein (LDL), and TG levels in host blood streams [12, 13]. Another study revealed that lipid uptake in hepatic stellate cells was provoked around trapped eggs [14]. Stanley et al. showed that lower cholesterol blood levels were caused by factors secreted by the schistosome eggs. Additionally, they observed agglomeration of cells with higher lipid contents in the outer parts of the granuloma [15]. The described processes in livers of infected animals and humans underline the importance of research regarding infection-induced metabolic changes.

Investigating lipids has become a highly active research area in the last years including instrumental advances, the detection of lipid biomarkers for various conditions, unraveling of double-bond positions in fatty acids, and the characterization of the lipidome of biological samples [16–19]. Although literature is available on the lipid composition of trematodes, the interaction of parasite and host has not been studied on the lipid level yet [20, 21]. Mostly, enzymes related to lipid metabolism have been investigated to speculate about their impact on lipid composition of worms and the host [22].

The parallel use of matrix-assisted laser desorption/ionization mass spectrometry imaging (MALDI MSI) and liquid chromatography tandem mass spectrometry (LC–MS/MS) is a widely used technique to investigate lipidomic profiles of samples while keeping the lateral information of the analytes. Commonly, lateral resolutions of about 10 µm are used [23]. This setup has already been applied to the analysis of *S.* *mansoni* worms [20, 24] and has now been optimized for egg-infected hamster liver tissue.

The combination of MALDI MSI and LC–MS/MS is the key to get a deeper understanding of the interactions between trapped eggs and the surrounding tissue. Analyzing infected tissue that still contains the parasites is beneficial to get an overview of the metabolic changes in both, the host tissue and the eggs. Due to the small size of the *S.* *mansoni* eggs of about 60–200 µm [25], a high lateral resolution of the applied MSI technique was essential. To the best of our knowledge, this is the first study of interactions of *S.* *mansoni* eggs (parasite) with the host liver tissue using MALDI MSI.

## Materials and methods

### Chemicals

A list of chemicals and suppliers is found in Table S1 in the Supplementary Information.

### Tissue samples

To keep the *S. mansoni* life cycle, *Biomphalaria glabrata* snails served as intermediate hosts and Syrian hamsters (*Mesocricetus auratus*) as final hosts [26]. The hamster model has been established because it is more permissive for schistosome infection compared to the mouse model, which requires more animals (reduction principle of the 3Rs). Both snails and hamsters were bred in-house (Biomedical Research Center Seltersberg, Giessen, Germany). All animal experiments were approved by the Regierungspraesidium Giessen (V54-19 c 20/15 h 02 GI 18/10 No. A 14/2017) and performed in accordance with the European Convention for the Protection of Vertebrate Animals used for experimental and other scientific purposes (ETS No. 123; revised Appendix A). For this study, liver samples of female hamsters were used. Three different groups of hamster livers were examined: non-infected hamsters were used as controls, hamsters infected with only one sex of *S.* *mansoni* cercariae (monosex-infected), and hamsters infected with both sexes of cercariae (bisex-infected) [26–28]. Each group consisted of three biological replicates. Bisex infections were carried out according to established protocols and were performed for 46 days, while monosex infections persisted up to 67 days [29] to ensure a sufficient quality of the worms. This especially applies for female schistosomes that need longer to grow and develop in hamsters without male partner. Non-infected controls were age-matched with bisex-infected hamsters. Liver samples were shock frosted in liquid nitrogen immediately after perfusion and subsequently stored at − 80 °C. Optical images of all measured samples can be found in Figure S14.

### OilRed staining

Neutral lipids were stained as described before [30]. Briefly, 8- to 10-µm cut-frozen sections were air-dried on glass slides, subsequently 10 min fixed in 10% formalin, briefly washed with running tap water 1–10 min, and afterwards rinsed for 2 s with 60% *iso*-propanol before staining with freshly prepared Oil Red O working solution (0.2% in 50% *iso*-propanol) for 15 min. After staining, slides were rinsed for 2 s with 60% *iso*-propanol, lightly stained with alum hematoxylin (5 dips), and rinsed with distilled water before mounting in aqueous mountant or glycerin jelly.

### MALDI MSI sample preparation

Fresh frozen liver samples were kept at − 80 °C prior to sample preparation. Cryosections of 20 µm thickness without any further fixation were prepared at − 25 °C using a Cryostat Microm HM 525 (Epredia, MI, USA). After cutting, sections were thaw-mounted onto common glass slides. Microscopic images were taken using a digital microscope (VHX-5000, Keyence, Neu-Isenburg, Germany). Sections were kept at − 80 °C until measurement.

For MS imaging analysis, samples were thawed in a desiccator for 30 min. For positive-ion mode, 2,5-dihydroxybenzoic acid (DHB) and for negative-ion mode, 1,5-diaminonaphthalene (DAN) were applied. 30 mg/mL of DHB was dissolved in acetone/H_2_O/trifluoroacetic acid (49.95:49.95:0.1, *v*:*v*:*v*). DAN was prepared at a concentration of 3.3 mg/mL in H_2_O/methanol (1:9, *v*:*v*). Matrix was applied using an ultrafine pneumatic sprayer (SMALDIPrep, TransMIT GmbH, Giessen, Germany) as described elsewhere [31]. In brief, 100 µL DHB and 400 µL DAN solution were applied with a flow rate of 10 µL/min and 30 µL/min, respectively. Nitrogen pressure was adjusted to 1 bar.

### MALDI MSI analysis

For MSI analysis, a high-resolution atmospheric-pressure MALDI imaging ion source (AP-SMALDI5 AF, TransMIT GmbH), coupled to an orbital trapping mass spectrometer (Q Exactive HF, Thermo Fisher Scientific (Bremen) GmbH, Bremen, Germany), was employed. Instrumental settings are listed in Table S2.

Mass accuracy of ± 1 ppm was maintained by internal calibration to a matrix cluster ion (*m*/*z* 716.12461 [5 DHB − 4 H_2_O + NH_4_]^+^).

### H&E staining

After MSI measurements, matrix was washed off with ethanol. Afterwards, sections were stained the following way:

At first, sections were rehydrated using 100%, 70%, and 40% ethanol and deionized water, 2 min each. Afterwards, samples were kept in hematoxylin solution for 12 min, followed by 10 min in tap water and 5 min in deionized water. After 1 min in eosin y solution, samples were dehydrated using deionized water, 40%, 70%, and 100% ethanol and xylene for 2 min each. Finally, samples were covered with Eukitt and a cover slip.

### LC–MS/MS sample preparation

For LC–MS/MS experiments, lipid extraction was performed according to Breitkopf et al. [32] with minor changes. In brief, 10 mg liver homogenate, 100 µL PBS buffer, and 350 µL methanol were vortexed for 1 min at 1500 rpm. Afterwards, 1 mL MTBE was added, and the mixture was shaken for 1 h at 20 °C and 1000 rpm. For better phase separation, 300 µL H_2_O was added, mixed for 1 min at 1000 rpm, and centrifuged for 8 min at 13,000 rpm. 1 mL of the organic upper phase was separated and dried under nitrogen flow. Dried samples were stored at − 80 °C until measurement. Prior to analysis, samples were reconstituted with 100 µL acetonitrile/*iso*-propanol/H_2_O (65:30:5, *v*:*v*:*v*). The whole extraction procedure was done additionally with 100 µL PBS as extraction blank; reconstitution buffer was used as blank. Samples obtained from different animals were used for LC–MS/MS analysis and for imaging experiments.

### LC–MS/MS analysis

Separation was performed on a UHPLC system (Ultimate 3000 UHPLC, Thermo Fisher Scientific) equipped with a reversed-phase 1.8 µm column (100 X 2.1 mm) (ACQUITY UPLC HSS T3, Waters GmbH, Eschborn, Germany). Mobile phase A consisted of H_2_O/acetonitrile (40:60, *v*:*v*), mobile phase B of *iso*-propanol/acetonitrile (90:10, *v*:*v*), both with 10 mM ammonium formate and 0.1 vol-% formic acid, as previously published [33]. Column gradient parameters for reversed-phase liquid chromatography separation are listed in Table S3. Flow rate was kept constant at 0.25 mL/min, injection volume was 10 µL. All samples of one group were measured consecutively. After each group, blanks were injected to clean the column. The gradient was used for both positive- and negative-ion mode.

After separation, tandem mass spectra were recorded using an orbital trapping mass spectrometer (Q Exactive HF-X, Thermo Fisher Scientific) for all samples. Heated electrospray ionization parameters are listed in Table S4, MS/MS parameters are listed in Table S5.

For quality control, pooled samples, containing 10 µL of all individual samples were added at the beginning, the middle and the end of the run. LC–MS/MS data were analyzed using LipidMatch Flow 3.1 [34]. To maximize the identification output, all samples were used as targets and ddMS2. Additionally, the equivalent carbon numbers (EQN) were calculated as EQN = CN − 2∙DB, with CN: carbon number of the fatty acids and DB: double bonds.

### Statistical analysis and MS image generation

Statistical analyses were performed using Perseus [35] and MetaboAnalyst [36] as described elsewhere [20]. For principal component analysis (PCA), data were not filtered, but row-wise normalized to constant sum. Afterwards, data were transformed by log10 normalization. For marker search, lipids found in bisex-infected samples were compared to monosex-infected and control samples, respectively. Therefore, measurements were grouped according to their biological sample group (control, monosex, bisex). For normalization, all signal intensities, expressed as peak areas, were divided by the total ion count of one sample. Values were standardized using *z*-scores. Afterwards, ANOVA tests with a permutation-based false discovery rate (FDR) of up to 5% were performed. Significant values were filtered and Tukey’s post hoc test was performed. The results were hierarchically clustered, leading to a list of significant “infection markers”.

These lists (for positive- and negative-ion mode) were used to generate MS images with Mirion [37]. Prior to image generation, no normalization or any other data processing was performed. Images were assessed manually for interesting distributions and overlaid according to their patterns. Identifications in the imaging data are only tentative. They were identified according to their exact mass by comparison with the list of markers identified by LC–MS/MS. Due to insufficient signal intensities, no MS/MS analyses were performed during the MSI measurements.

## Results and discussion

### Granuloma around eggs in liver tissue of infected animals exhibits an accumulation of lipids

**Fig. 1:**
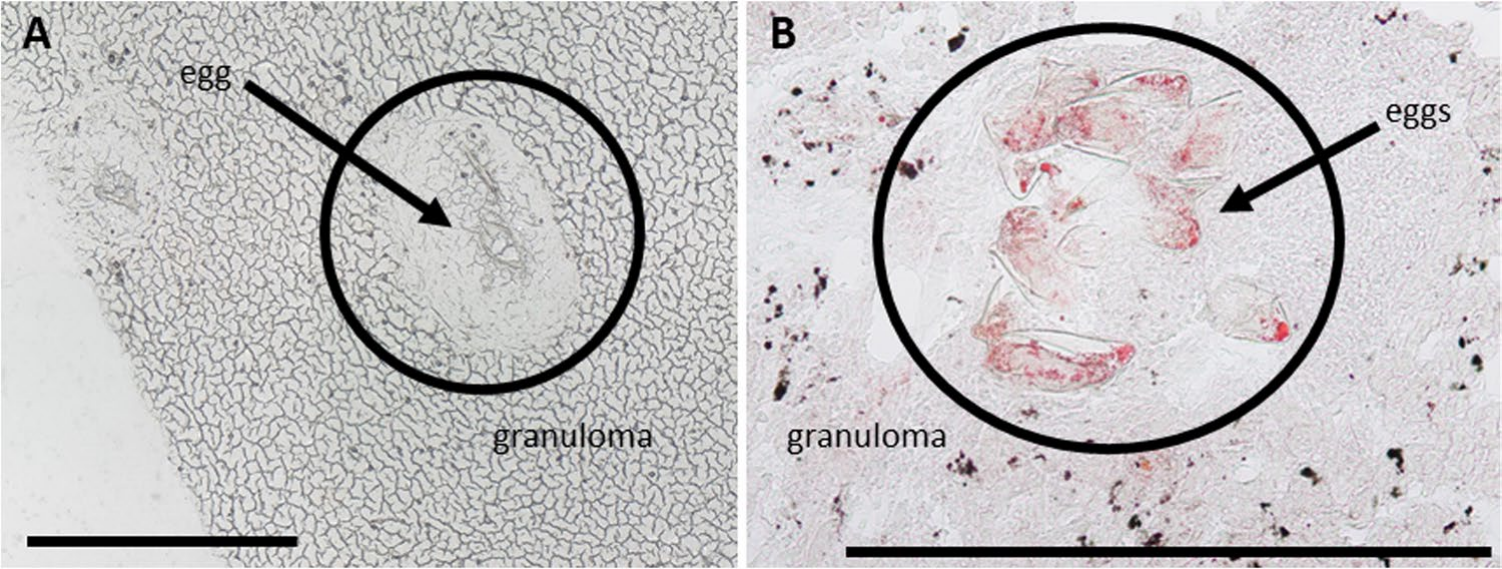
Microscopic images of eggs with granuloma in livers of bisex-infected hamsters. (**A**) Light microscopic image of unstained tissue. (**B**) Tissue stained with oil red. Lipid accumulation in granuloma is clearly visible. Scale bars are 500 µm

It is well known that schistosome infection leads to granuloma formation around eggs deposited in tissues such as the liver [7]. Granuloma mainly consist of macrophages and other immune cells [9, 10], which are assumed among others to play important roles in providing nutrients in form of lipids for the eggs [11]. On the other hand, they shield other hepatocytes from injury [38]. Granulomas are readily visible in the microscopic images of prepared liver sections as shown in Fig. 1A. Staining with oil red revealed an accumulation of neutral lipids in the granuloma as shown in Fig. 1B.

To distinguish between effects of the eggs and systemic effects of worm infection, bisex-infected samples were compared to monosex-infected samples, which are expected to produce no eggs. In rare cases, however, non-fertilized egg-like structures appear in hamsters infected with clonal female cercariae (monosex-infected), which can be accompanied by other types of host reactions [39]. For our studies, only monosex-infected samples without eggs were included. Non-infected samples were defined as controls to differentiate between healthy (no eggs) and infected (eggs) samples. Microscopic images of the three different sample types used in this study are shown in Figure S1 in the supplementary file. While a detailed histological investigation of the involved cell types in the immune response of the hamster is not the scope of this study, further information on the topic will be found in the literature [von Bülow et al. 2022, in preparation]. Granulomas were formed due to trapped eggs in the bisex-infected (C) samples. The *S.* *mansoni* eggs are clearly visible within the granulomatous area in the zoomed-in image (D) of the bisex-infected samples. Tissue areas that were subsequently subjected to MS imaging experiments were selected based on microscopic images to ensure granuloma inclusion in case of bisex samples.

During further analysis, microscopic images were used to allocate identified markers to infection-specific morphological structures, such as eggs and granuloma. While overlaying optical and MS images, marker localization was confirmed. Due to the high thickness of tissue sections used for MALDI (≈ 20 µm), histological investigation is hindered. Moreover, tissue sections can only be stained after the MSI experiment, and cellular structures are affected and partly destroyed by the laser irradiation.

An exemplary microscopic image of an H&E-stained section after MSI analysis can be found in Figure S2.

### LC–MS/MS analysis detected infection markers

**Fig. 2:**
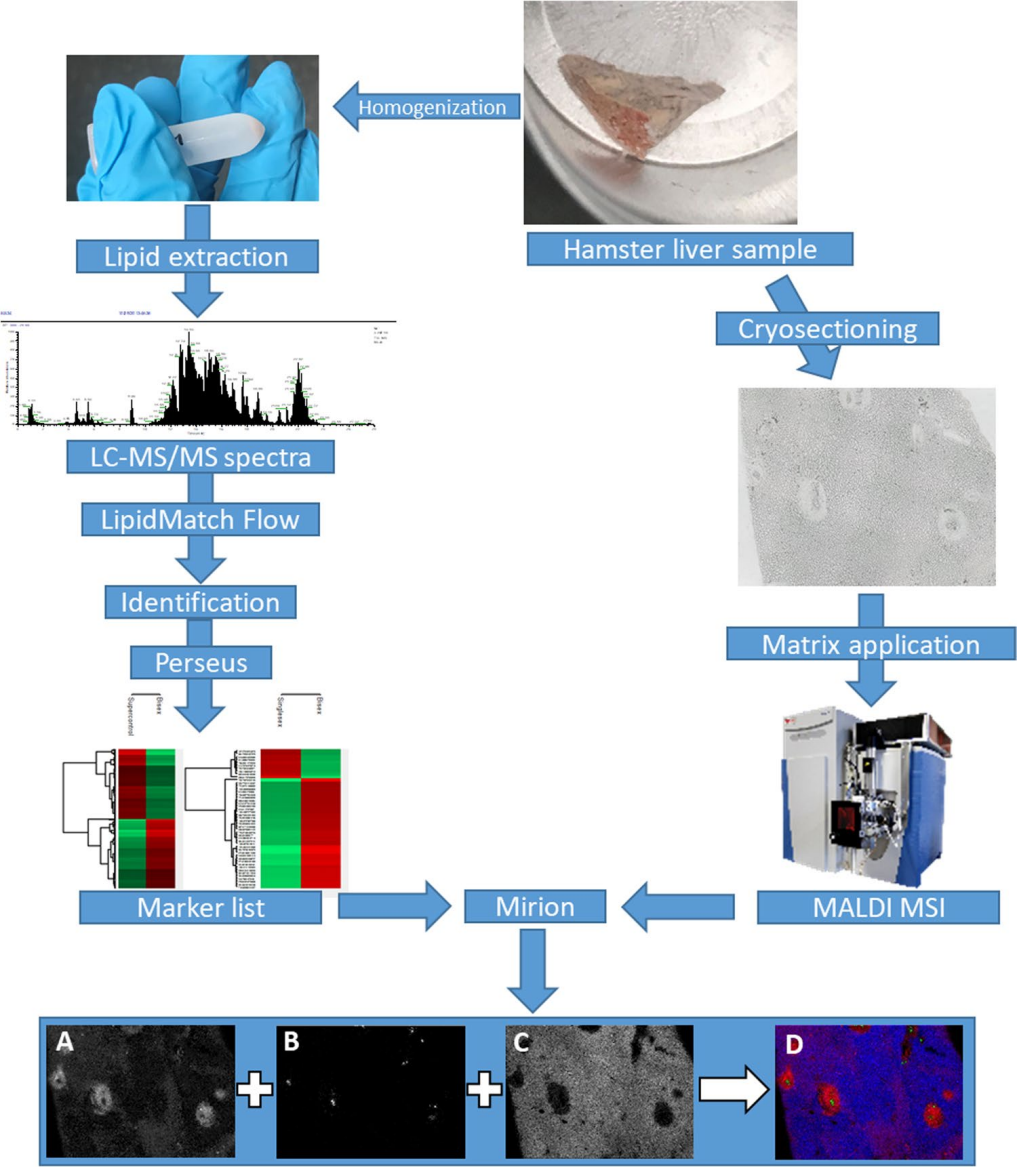
Hamster liver samples were either homogenized or cryosectioned. After homogenization, lipids were extracted, and samples were analyzed using LC–MS/MS. With the help of LipidMatch Flow and Perseus, identified signals were annotated and markers were identified that occurred with statistically determined significance. After cryosectioning, matrix was applied, and samples were measured using MALDI MSI. Using the software Mirion and the marker list from LC–MS/MS experiments, MS images were generated: (**A**) m/z 500.275684, LPE(20:4), [M − H]^−^; (**B**) m/z 866.592639, PS(42:4), [M − H]^−^; (**C**) m/z 776.526946, MMPE(16:0_22:6), [M − H]^−^; (**D**) RGB overlay of MS images A, B, C

In order to identify markers for *S.* *mansoni* infection and to determine their distributions in the tissue, we combined different mass spectrometric techniques. Experiments were performed with three sample groups (non-infected, monosex-infected, and bisex-infected), comprising three biological replicates of each group. An overview of the workflow is given in Fig. 2. The top right part shows a piece of hamster liver that was used for analysis. Following the figure counterclockwise, parts of liver samples were homogenized followed by lipid extraction. Applying LC–MS/MS, 372 significantly occurring markers were found in the extracts after lipid identification in positive- and negative-ion mode with LipidMatch Flow and statistical analysis of the data with Perseus. An exemplary LC–MS/MS spectrum can be found in Figure S3. All lipid identifications are based on head group and fatty acid fragments with one exception that was only confirmed by class. Using MetaboAnalyst, PCA was performed. As the scores plot in Figure S5 shows, the three sample groups were well separated based on LC–MS/MS data, already suggesting that the lipid profiles vary significantly. Additionally, the PCA shows all three technical replicates of the pool samples arranged in very close vicinity, indicating that the LC measurements were acquired with stable performance.

In parallel, cryosections of the liver samples were prepared and analyzed in positive- as well as in negative-ion mode with MALDI MSI, as shown in Fig. 2. For some of the infection markers, distribution patterns matching with optical images were found by annotating MSI data using the marker list from LC–MS/MS experiments in Mirion. The three native color channels (red, green, and blue) were used to overlay the three selected MS images (D), showing significantly different spatial distributions for the three markers. Comparison with the microscopic image revealed their connection to different compartments of the inflammation. All identified markers and their predominant localization (if applicable) are listed in an additional excel sheet (see supplemental information, “infection marker list”).

The overlap between the detected *m*/*z* data sets of LC–MS/MS and MALDI MSI was not perfect. Most of the detected *m*/*z* values were found with only one method. This effect is due to the different ionization mechanisms of ESI and MALDI that favor ionization of different lipid classes as well as the formation of different adduct species. While the solvent in LC–MS/MS experiments contained ammonium formate, and predominantly [M + NH_4_]^+^ ions were formed, for example, for triglycerides, the same lipid species were detected as alkali metal adducts with MALDI MSI. To enhance the overlap of the two data sets, the *m*/*z* values of the identified lipids in LC–MS/MS were re-calculated. In case of triglycerides, *m*/*z* values for [M + H]^+^, [M + Na]^+^, and [M + K]^+^ ions were calculated and were used to create images. An example can be found in Figure S6 and is discussed later in the MSI part. Another aspect that has to be kept in mind is the difference in sample preparation. While samples for LC were homogenized and lipid extraction was performed, whole tissue sections were used for MSI experiments. Therefore, matrix effects might play an important role regarding comparability of MSI data to LC–MS/MS data. Matrix effects are present also for LC measurements but should be smaller compared to MSI. Furthermore, some markers detected in MSI measurements might be absent in LC data due to low solubility.

**Fig. 3:**
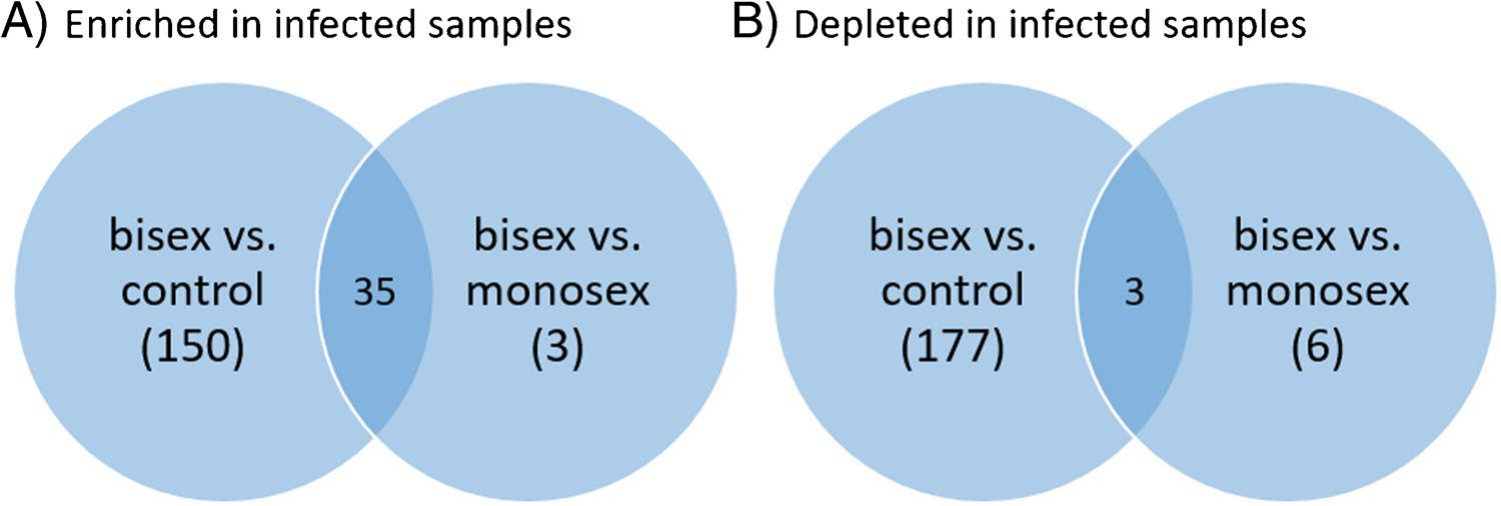
Venn diagram showing the numbers of infection markers found. Many more infection markers were found to be enriched when comparing bisex-infected with control samples than with monosex-infected samples (**A**). The same holds for depleted infection markers (**B**). The terms “enriched” and “depleted” have to be used carefully since further biological analysis is needed to verify that the eggs actually take up and metabolize the lipids. Here, the terms are meant to describe changes in signal intensities found in LC–MS/MS measurements

More markers distinguish between bisex-infected and control samples than between bisex- and monosex-infected samples, as seen in Fig. 3. Markers found for monosex-infected samples were mostly those also found for bisex-infected samples (35 “enriched” markers in Fig. 3). The effects of *S. mansoni* infection on the lipid composition of the host liver tissue are manifold. Although infection with monosex cercariae does not result in egg deposition and granuloma formation, the unpaired parasites still provoke the host organism and the cercarium develops into an adult worm that resides in different organs of the host. In a previous study, up to 85% of the unpaired female worms and 65% of male worms were found in mouse liver after 8 weeks. The female worm induced an accumulation of inflammatory cells in blood vessels and the surrounding liver tissue [40–42]. These effects also play a role when paired worms from a bisex-infection are present, but morbidity-inducing liver damage is ultimately caused by the deposited eggs. Altogether, the hepatic lipid composition of bisex-infection shows more similarities to a monosex-infection than to the control which might reflect the common changes due to worm presence in both infection states.

**Fig. 4:**
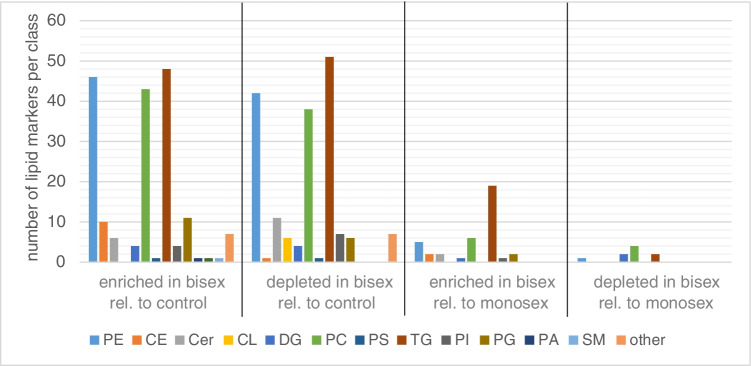
Enrichment and depletion of lipids of several lipid classes in bisex-infected hamster liver, relative to non-infected controls or monosex-infected hamsters. Significantly more enriched/depleted markers were observed relative to controls than to monosex-infected samples. Markers were found by comparing the groups of bisex-infected samples to monosex-infected or control samples. No individual comparison between the three samples of one sample group was performed. Lipids with a FDR of up to 5% were assumed as infection markers

Changes in lipid composition were mostly found in the groups of PCs, PEs, and TG (Fig. 4). All subgroups of one major phospholipid class were summed in the same group. As an example, all PEs, lyso-PEs, oxidized PEs and oxidized lyso-PEs were summed up and shown in the PE bar (for full information on the lipid species detected, please refer to the “Infection marker” table in the Supplementary information). Interesting lateral distributions of such subgroups are discussed in more detail below in the MS imaging part. Both enrichment and depletion were found due to egg deposition. The terms “enriched” and “depleted” have to be used carefully here, since further biological analyses are needed to verify that the eggs take up and metabolize the depleted lipids. Until then, the terms are meant to describe changes in signal intensity during LC–MS/MS measurements with no information about depletion or enrichment location.

The majority of markers belong to the TGs, followed by PEs and PCs. These lipid classes are known for their good detectability in LC–MS measurements. We calculated the average number of carbon atoms and double bonds in the detected PC, PE, and TG markers for comparison between the sample groups. While no striking difference was found for PC and PE markers, triglycerides revealed a varying number of double bonds in the fatty acid chains. The 48 TG markers that were found enriched in bisex-infected samples compared to control had an average number of double bonds of *n* = 8.4 while the 51 TG markers depleted in bisex-infected samples compared to control samples had an average double bond number of *n* = 3.1. The carbon number differed only in the range of the standard deviation. By examining the “Infection marker” table in the Supplementary information, one can find no arachidonic acid (AA, 20:4) or docosahexaennoic acid (DHA, 22:6) in the markers of the control samples, but one or both of these fatty acids appear in 52% of TGs enriched in bisex-infected samples. It was shown previously that AA treatment of hamsters infected with *S. mansoni* reduced worm burden and the number of eggs [41], so the presence of AA in bisex-infected samples might be a protective response of the immune system. The treatment with DHA also resulted in reduced worm burden and lower egg count, but the effect was not as clear as for AA.

Enrichment was found in LC–MS/MS measurements for all cholesterol ester markers (CE), while none of the CE signals was found to be depleted relative to control samples. Cardiolipin markers (CL), on the other hand, were found depleted, while no detected CL was enriched relative to control samples. No characteristic distribution patterns were found for these species (CE and CL) in MALDI MSI measurements, which is either due to a missing lateral specificity or an insufficient signal-to-noise ratio in MALDI for these compounds at small spot sizes (high lateral resolution).

### Lipid distributions revealed by MALDI MSI

**Fig. 5:**
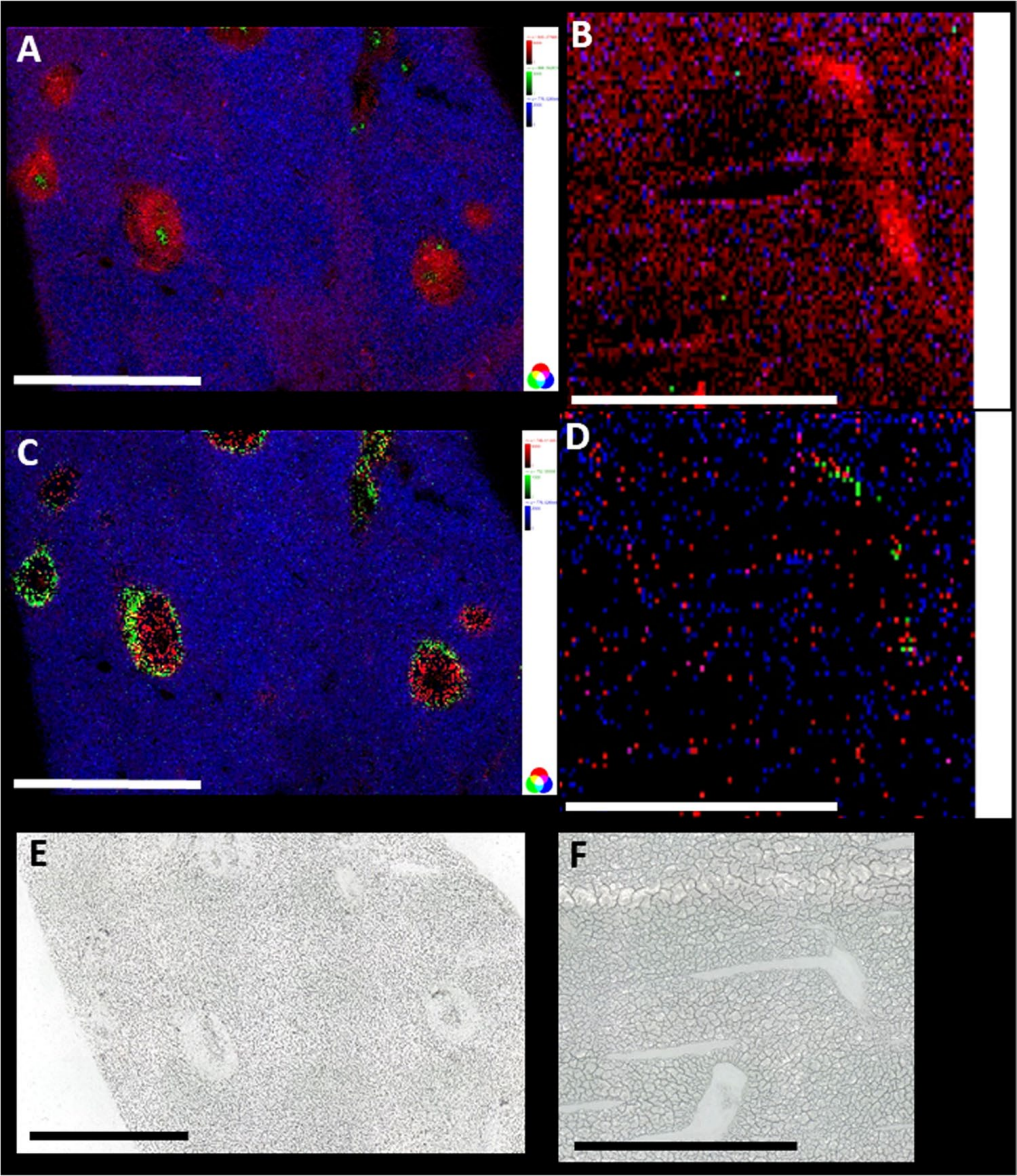
Comparison between liver sections of a bisex-infected hamster (**A, C**) and a healthy control sample (**B, D**). For A, m/z 500.275684, LPE(20:4), [M − H]^− ^(red) was found to be a marker for granuloma; m/z 776.526946, MMPE(16:0_22:6), [M − H]^−^ (blue) a marker for surrounding tissue; and m/z 866.592639, PS(42:4), [M − H]^− ^(green) a marker for schistosome eggs. For the non-infected sample, B shows the same ions in the same colors, but no characteristic distribution was found. This underlines that statistical markers found by LC–MS/MS also show recognizable and allocatable distributions in the imaging measurements. In image C, m/z 746.511353, plasmenyl-PE(P-16:0/22:6), [M − H]^− ^(red) as a marker for granuloma and m/z 752.555581, plasmanyl-PE(O-18:0/20:4), [M − H]^− ^(green) as a marker for granuloma borders were selected for creating the RGB image. Again, m/z 776.526946 was taken as a marker for non-affected tissue (blue). The same coding as in C was chosen for image D of a non-infected (control) sample, and again, we found no characteristic distribution. Scale bars are 1 mm. Single-ion images are shown in Figure S7 and Figure S8. **E** and **F** are the corresponding optical images of the samples

In Fig. 5A and C, lateral distributions of selected markers defined in our LC–MS/MS experiments are shown. For all images, MMPE(16:0_22:6) (monomethylphosphatidyl ethanolamine) was chosen to visualize the liver tissue in blue. This signal was only observed in the non-affected tissue surrounding the granuloma. The corresponding ion was found as a marker that is enriched in bisex samples compared to control samples. Inside the granuloma, lysolipid metabolites of PE were found (red). Images B and D, obtained from non-infected samples, do not show any localized enrichment of these markers that can be attributed to tissue structures. This suggests that the eggs might accumulate certain lipid species, for example, the tentatively identified PS(42:4) (Fig. 5). On the other hand, they appear to consume and thus downgrade the contents of certain lipids in the close vicinity of granulomas in bisex samples, as, for example, for MMPE (see single-ion images and optical images in Figure S7, S8, and S14). Taking a look at the single-ion images, “holes” in the distribution of granuloma-specific ions were observed. The microscopic images show that this is not due to missing tissue but schistosome eggs are found at these positions. This is a further hint that the eggs take up these lipids and metabolize them. However, as no labelled tracer studies were carried out, this is only a hint and not a clear proof. A deeper look into lipid uptake of schistosome eggs is described in a recent study of von Bülow et al. [in preparation]. Of special interest are the two lipids shown in the green color channel in Fig. 5A and C, which were found in distinct areas of the granuloma only. The compound at *m*/*z* 752.555581 was identified by LC–MS/MS as a PE lipid (plasmanyl-PE(O-18:0/20:4), [M − H]^−^); the other compound at *m*/*z* 866.592639 was tentatively assigned as PS(42:4), [M − H]^−^. Other examples can be found in Figure S9. While some marker lipids were found within or in direct contact to the eggs, others were located mostly in the outer regions of the granuloma (shown in the green color channel). Eggs have to take up nutrients from their host tissue to survive [43] and our MS image data seem to substantiate this assumption. The enrichment or depletion of specific lipids might be related to the presence of cells forming the granuloma and therefore displacing the hepatocytes. We found no clear correlation between localization and an entire lipid class but only with individual lipid species.

Additionally, the distribution pattern based on different adducts for the same lipid was investigated. This can be exemplarily seen in Figure S6. Here, the distribution of PC(O-34:1) is shown as proton, sodium, and potassium adducts. In general, this lipid was mainly found in the granuloma around but not in the eggs. However, on closer examination, signal intensities and localization outside the granuloma region vary slightly. This can be due to different distributions of the alkali metals in the tissue. In general, signal intensities of some lipid classes (e.g., TGs) were not that high. After dividing low abundance of one compound to three different adduct signals, intensities might be too low for detection. Therefore, not all adducts were found for each lipid.

We have chosen the lipid class of PCs to take a deeper look at the distributions of several lipid species. Interestingly, different distribution patterns were found. For example, PC(16:0_20:5), observed at *m*/*z* 802.535727 as [M + Na]^+^, was found to be evenly distributed in the whole sample except in the granulomatous areas in liver samples of bisex-infected hamsters, where it was depleted (Figure S10). In contrast, PC(19:0_20:2), detected at *m*/*z* 850.629627 as [M + Na]^+^, was found to be enriched in granulomas (Figure S11). PC(16:0_18:0), detected at *m*/*z* 800.556617 as [M + K]^+^, was found only in the granulomas and just rarely in the surrounding tissue or in not-infected samples (Figure S12). The according mass spectrum and extracted ion chromatogram are shown in Figure S4. PC(20:0_20:3), detected at *m*/*z* 862.630305 as [M + Na]^+^, showed some enrichment in and directly around the eggs, but not in the rest of the sample (Figure S13). These observations provide first hints about possible metabolic changes in the tissue due to infection.

**Fig. 6:**
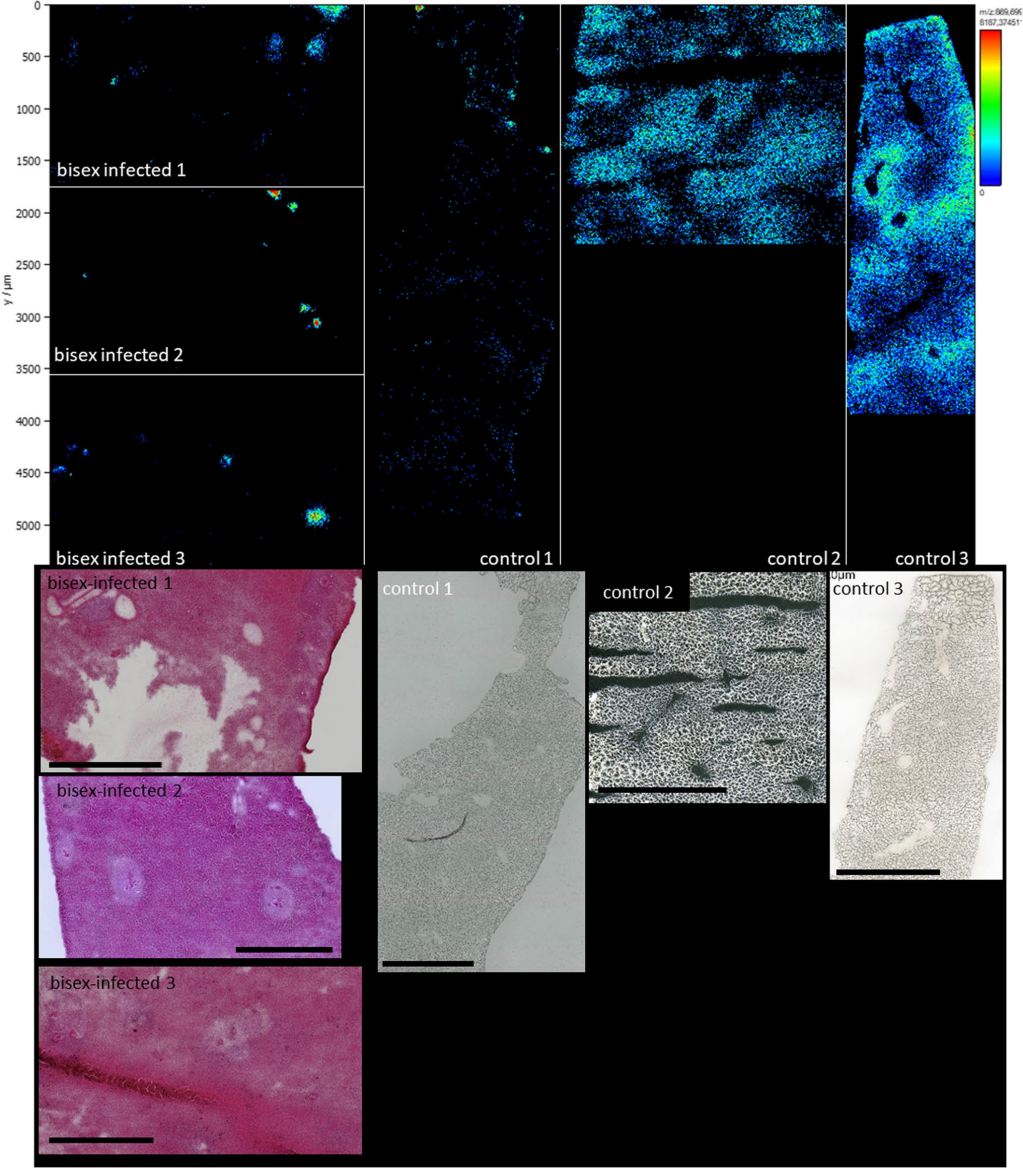
Upper part: Distribution comparison of m/z 869.698690 TG(16:0_16:1_18:1) [M + K]^+^. Liver samples of bisex-infected hamsters are shown on the left side; control samples are grouped on the right side. While the lipid is evenly distributed in the control samples, accumulation in the eggs and the surrounding granuloma area of the bisex sample group is clearly visible. Overall, this triglyceride was found enriched in control samples by LC–MS/MS. Lower part: Corresponding optical images of the samples. Scale bars are 1 mm

Another example is shown in Fig. 6. Here, the lateral distribution of TG(50:2), detected as [M + K]^+^ ion at *m*/*z* 869.69869, is shown. This lipid was detected at *m*/*z* 848.771097 as [M + NH_4_]^+^ in LC–MS/MS experiments and also found in eggs before by Giera et al. [21]. Our statistical LC–MS/MS analysis revealed that this TG was depleted in tissue of bisex-infected hamsters. MSI data revealed distribution changes due to infection. While the lipid was found to be evenly distributed in the three control samples, it was only found in the granuloma of the bisex-infection group. All images shown in Fig. 6 were adjusted to the same signal intensity. Therefore, pixel brightness in all sub-images corresponds to the same signal intensity scale. Bisex samples on the left-hand side show negligible signal intensities of TG(50:2) except from the granulomatous areas, where signal intensities peaked to NL = 8*10^3^. In the control samples, the lipid was found ubiquitously and signal intensities were in the range of NL = 1*10^3^ to NL = 2.2*10^3^. In control sample 1, signal intensities were lower compared to the other control samples, but the signal was still evenly distributed throughout the whole tissue section.

This tendency was also observed for eight other triglycerides. Due to low signal intensities, however, the effect was not as obvious in some cases. For the triglycerides with the lowest signal intensities, accumulation of the respective lipid was observed in the granuloma of the bisex-infection group, whereas detection in control samples was limited to only a few pixels per sample. Interestingly, we have not observed the opposite trend of a depletion in granuloma for any triglyceride in our MSI data. These data provide new insights into the localization and accumulation of triglycerides in livers of *Schistosoma*-infected hamsters at the level of individual lipid species.

We also compared infection markers found in our study with those found in schistosome eggs by Giera et al. [21]. Tentative markers that we specifically found in eggs (DMPE(18:0_22:5) and PS(42:4)) were not detected by Giera et al. or Kadesch et al., neither in eggs nor in schistosome worms. Infection markers of the lipid classes CE, LPC, PC, and TG were in good accordance with previous data [20, 21], suggesting that the eggs might take up the respective lipids, perhaps as nutrients. Other markers, such as PEs, CLs, dimethylphosphatidylethanolamines (DMPEs), and diglycerides (DGs), seemed to be characteristic for the host tissue since they were not detected in eggs by Giera et al.

Additional investigations are needed to get a better understanding of the underlying mechanisms of lipid localization, presumptive uptake mechanisms, and accumulation. These might be related to lipid consumption by the eggs or to reactions of the host’s immune system. Other studies have shown that granuloma protects the host from enzymes secreted by the eggs [7, 44], and the identified lipids in the outer regions of the granuloma might be part of this defense mechanism. Stanley et al. reported about lipid-enriched cells in the outer part of the granuloma and suggested that immune cells, such as macrophages, might play an important role in the changes of the lipid profile of the host’s liver [15]. According to our findings, only plasmanyl- and plasmenyl-PEs (plasmalogens) seemed to accumulate at the borders of granuloma (see Fig. 5). Plasmalogens were mainly found in the outer tegumental membranes of adult worms [45]. This membrane is the contact region between host and parasite and plays an important role in defending the parasite against the host’s immune system [46, 47].

The results of our study provide first insights into the alteration of lipid profiles in hamster livers after infection with *S.* *mansoni* and egg deposition. Especially the detailed information on lipid species level that was achieved by LC–MS/MS analysis in connection with lateral information gained through MALDI MSI will open novel perspectives to unravel the roles of individual lipid species and lipid classes in the context of the egg-induced pathological changes in the host liver tissue.

## Conclusion

With the help of mass spectrometric techniques, we characterized morphological changes, such as granuloma formation in the liver tissue of *S.* *mansoni*-infected hamsters at a molecular level. Alterations in the lipid profile were examined by LC–MS/MS of homogenized samples, and for the first time, we identified markers specific for infection. These markers were additionally analyzed by MALDI MSI, exhibiting their distribution in tissue sections. The established protocol allowed not only to visualize and analyze entire granulomas based on selected markers but also to indicate substructures inside the granulomas. Furthermore, we provided evidence for a specific distribution of individual lipid species and their enrichment or absence in tissue sections containing parasite eggs. It is tempting to speculate about a parasitic role also for the egg stage of *S.* *mansoni*, which might acquire lipid resources from its host tissue environment. Further studies will address presumptive uptake mechanisms and the role of these lipids in the host-parasite interaction of *S.* *mansoni*.

## Supplementary Information

Below is the link to the electronic supplementary material.

Supplementary file1 (PDF 19.9 MB)

Supplementary file2 (XLSX 96 KB)

## Abbreviations

AA, 20:4: Arachidonic acid
CL: Cardiolipin
CE: Cholesterol ester
DAN: 1,5-Diaminonaphthalene
DG: Diglyceride
DHB: 2,5-Dihydroxybenzoic acid
DMPE: Dimethylphosphatidylethanolamine
DHA, 22:6: Docosahexaennoic acid
ECN: Equivalent carbon number
FDR: False discovery rate
LC–MS/MS: Liquid chromatography tandem mass spectrometry
LDL: Low-density lipoprotein
LPE: Lysophosphatidylethanolamine
MALDI MSI: Matrix-assisted laser desorption/ionization mass spectrometry imaging
MMPE: Monomethylphosphatidylethanolamine
NTDs: Neglected tropical diseases
PC: Phosphatidylcholine
PE: Phosphatidylethanolamine
PS: Phosphatidylserine
PZQ: Praziquantel
PCA: Principal component analysis
So: Sphingosine
TG: Triglyceride
