# Supplementary material for: Changes in the lipid profile of hamster liver after Schistosoma mansoni infection, characterized by mass spectrometry imaging and LC–MS/MS analysis
Source: Anal Bioanal Chem. 2022 Mar 23;414(12):3653–65. doi: 10.1007/s00216-022-04006-6 (PMC9035427; doi:10.1007/s00216-022-04006-6)
Supplement: Supplementary file 1 — Supplementary file1 (PDF 19.9 MB) [file 216_2022_4006_MOESM1_ESM.pdf]

Supplementary information

Changes in the lipid profile of hamster liver after *Schistosoma mansoni* infection, characterized by mass spectrometry imaging and LC-MS/MS analysis

Katja R. Wiedemann[1], Alejandra Peter Ventura[1], Stefanie Gerbig[1], Martin Roderfeld[2], Thomas Quack[3], Christoph G. Grevelding[3], Elke Roeb[2], Bernhard Spengler[1#]

[1]Institute of Inorganic and Analytical Chemistry, Justus Liebig University Giessen, Giessen, Germany
[2]Gastroenterology, Justus Liebig University Giessen, Giessen, Germany
[3]Institute for Parasitology, Justus Liebig University Giessen, Giessen, Germany

Author to whom correspondence should be addressed
e-Mail address: bernhard.spengler@anorg.chemie.uni-giessen.de

Chemicals

Table S1: Used chemicals and their specifications.

Chemical name	Quality grade	manufacturer
**1,5-diaminonapthalene** (DAN)	97%	Acros Organics, Geel, Belgium
**2,5-dihydroxy benzoic acid** (DHB)	for synthesis	Merck, Darmstadt, Germany
**2-propanol**	for HPLC	Chemsolute, Renningen, Germany
**acetone**	HiPerSolv	VWR International, Fontenay-sous-Bois, France
**ammonium formate**	99.995%	Sigma-Aldrich, Steinheim, Germany
**acetonitrile**	HiPerSolv	VWR International, Fontenay-sous-Bois, France
**Eosin Y solution**		Sigma-Aldrich, Steinheim, Germany
**ethanol**	Uvasol	Merck, Darmstadt, Germany
**Eukitt quick hardening medium**		Sigma-Aldrich, Steinheim, Germany
**formic acid**	for mass spectrometry	Honeywell, Morris Plains, NJ, USA
**Mayer's hematoxylin solution**		Sigma-Aldrich, Steinheim, Germany
**methanol**	LiChroSolv	Merck, Darmstadt, Germany
**Methyl-tert-butylether (MTBE)**	for HPLC	Sigma-Aldrich, Steinheim, Germany
**Oil Red**		Sigma-Aldrich, Steinheim, Germany
**paraformaldehyde**		Roth, Karlsruhe, Germany
**phosphate buffered saline (PBS)**		Gibco, Carlsbad, CA, USA
**trifluoro acetic acid**	Uvasol	Merck, Darmstadt, Germany
**water**	HiPerSolv	VWR International, Fontenay-sous-Bois, France
**xylene**	for analysis	Merck, Darmstadt, Germany

MALDI MSI measurements

Table S2: Settings used for data acquisition with MALDI MSI.

Parameter	Setting
m/z	250-1000
**Ion mode**	Positive and negative
**Resolution**	240,000 at *m/z* 200
**Ion injection time**	500 ms
**Scan rate**	1 / s
**Spray voltage**	3 kV
**Capillary temperature**	250°C
**Lock mass**	*m/z* 716.12461 $[5\ DHB - 4\ H_2O + NH_4]^+$, none for DAN
**Pixel size**	10 µm

Mode	2D pixel mode
20% filter	On
Attenuator	15-20°

LC-MS/MS measurements

Table S3: LC gradient.

Time / min	Mobile phase A / %	Mobile phase B / %
0	70	30
2	57	43
2.1	45	55
12	35	65
18	15	85
20	0	100
25	0	100
25.1	70	30
28	70	30

Table S4: Source parameters for LC-MS measurements.

Parameter	Positive-ion mode	Negative-ion mode
Sheath gas / a.u.	40	45
Auxiliary gas / a.u.	15	12
Sweep gas / a.u.	2	1
Spray voltage / kV	3.5	3.5
Capillary temperature / °C	300	320
S-lens RF	50	55
Auxiliary heater temperature / °C	300	320

Table S5: MS parameters for LC-MS measurements, in () for MS/MS.

Parameters	Positive-ion mode	Negative-ion mode
Run time / min	28	28
Internal lock mass	391.28421	-
Charge state	1	1
Exclusion list [47]	On	-
Resolution	70k (35k)	70k (35k)
AGC target	10^6 (10^5)	10^6 (10^5)
Maximum injection time / ms	250 (75)	250 (75)
Scan range	200-1800	200-1800
Top N	15	15
Isolation window / *m/z*	1	1
Stepped NCE	25, 30	20, 30, 40
Underfilll ration / %	0.6	0.6
Intensity threshold	$8 \cdot 10^3$	$8 \cdot 10^3$
Exclude isotopes	On	On
Dynamic exclusion / s	8	8

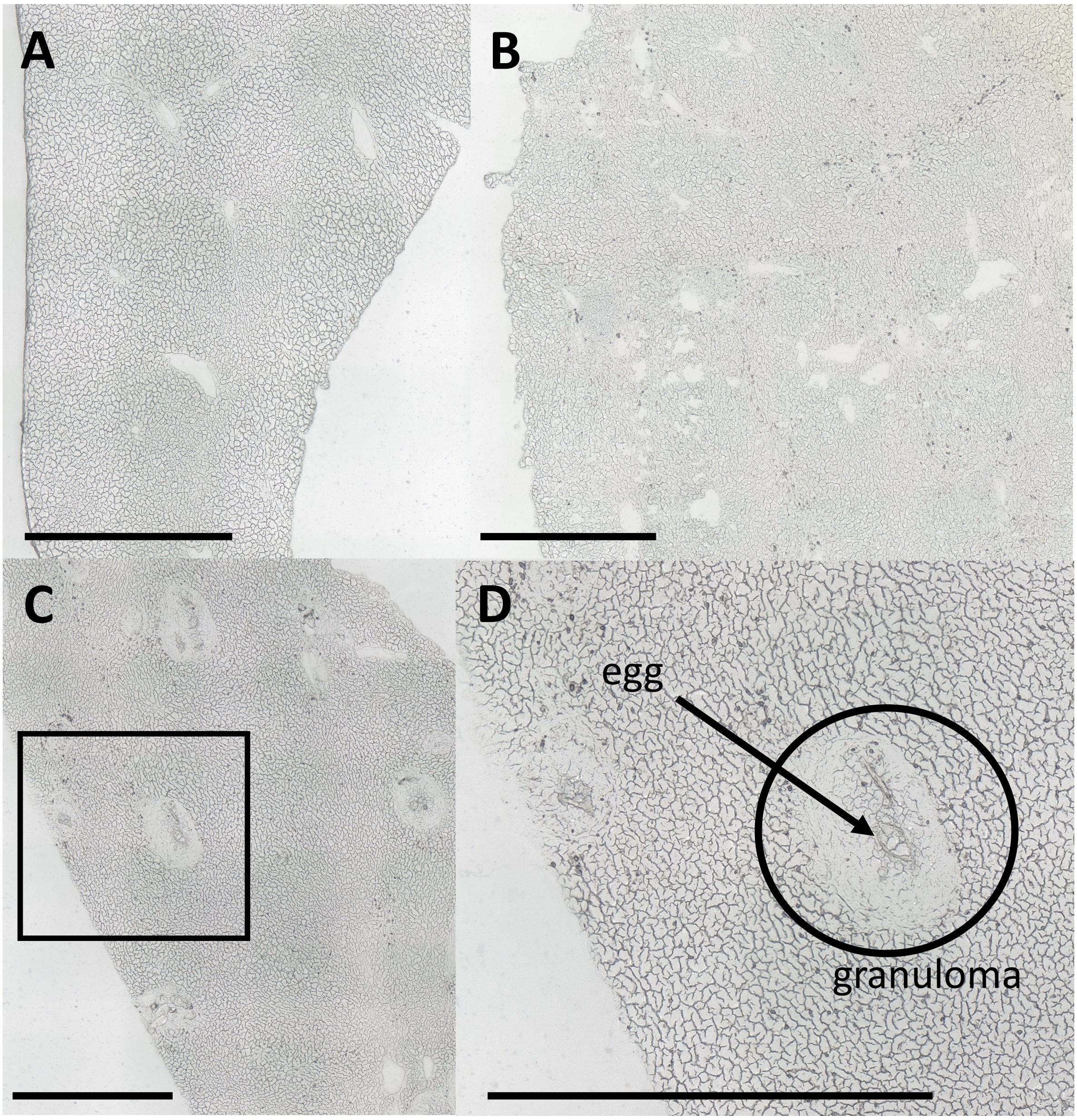

Figure S1: Light microscopic images of hamster liver cryosections of 20 µm thickness. (A) Non-infected, (B) monosex-infected, (C) bisex-infected, (D) zoomed area of (C), showing a nested egg and inflammation area around egg; scale bars are 1 mm.

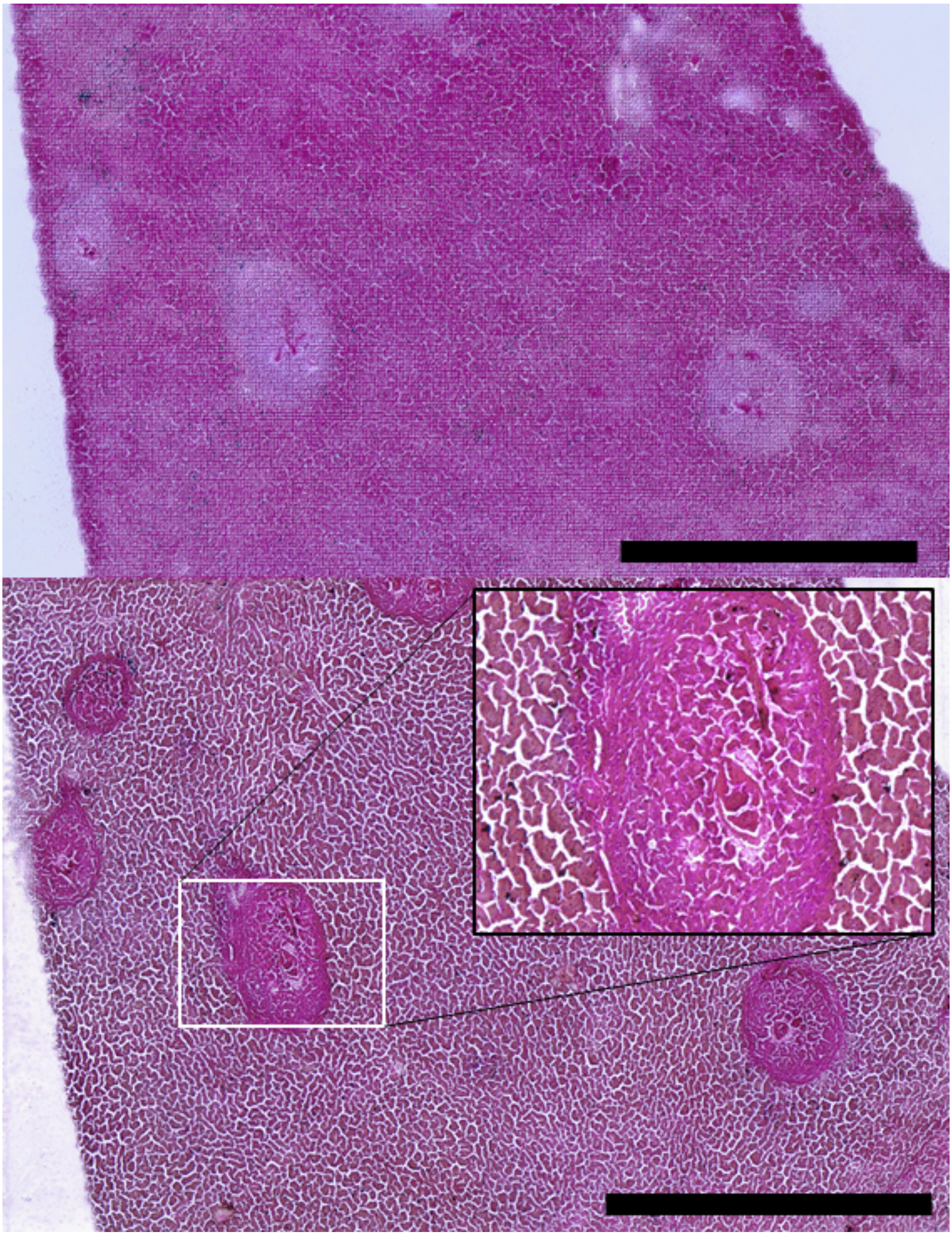

Figure S2: H&E stained sections of bisex-infected sample 2. Staining was performed after MSI measurements, therefore laser ablation spots are clearly visible. However, granuloma can still be identified around the eggs.

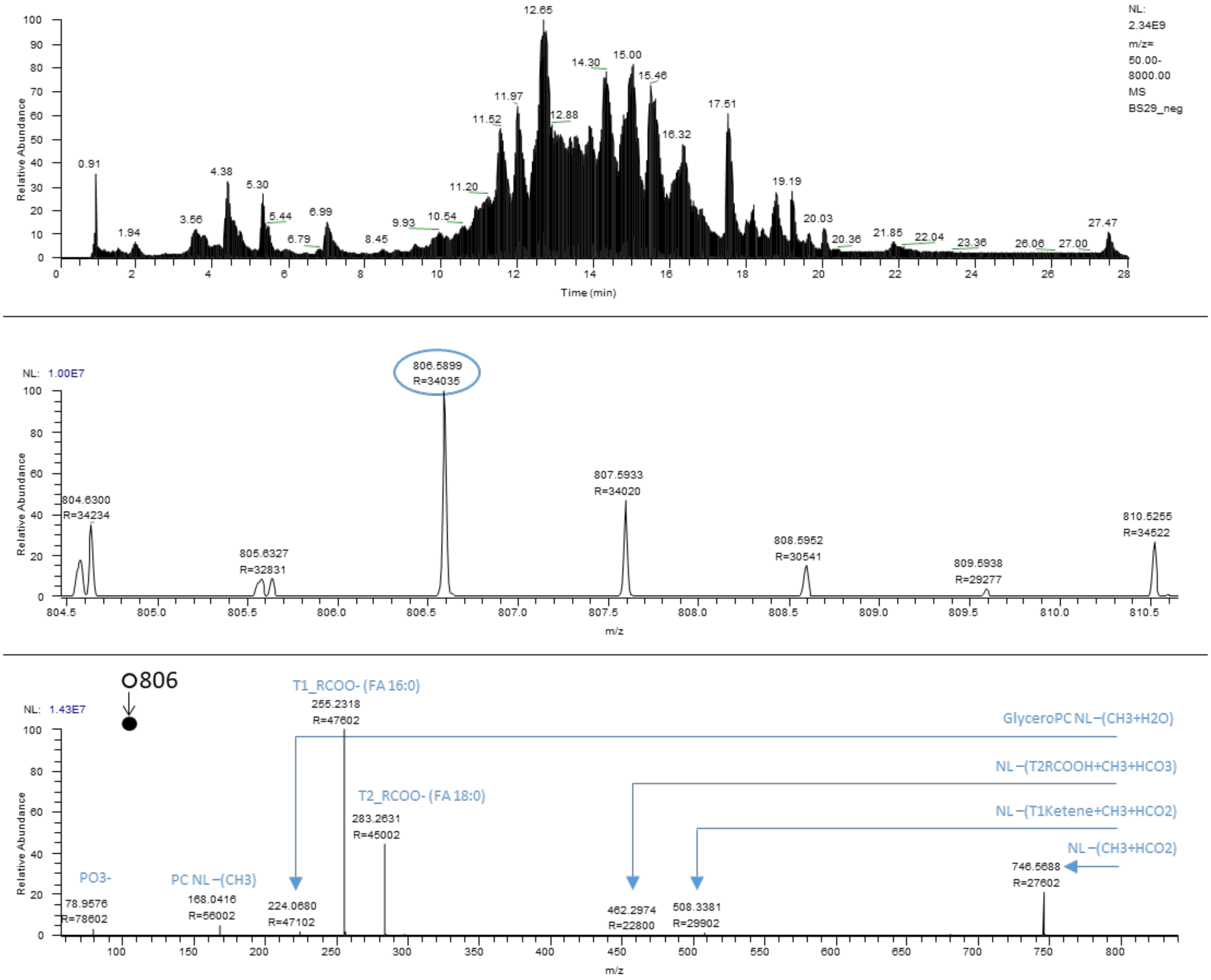

Figure S3: Exemplary LC-MS/MS spectra. In the upper part, the chromatogram of the LC separation is shown. In the middle, a zoom of the full MS spectra can be found. The peak of interest is marked with a blue circle. The lower part shows the MS/MS spectra of the peak of interest. Fragment ions and losses are stated in blue.

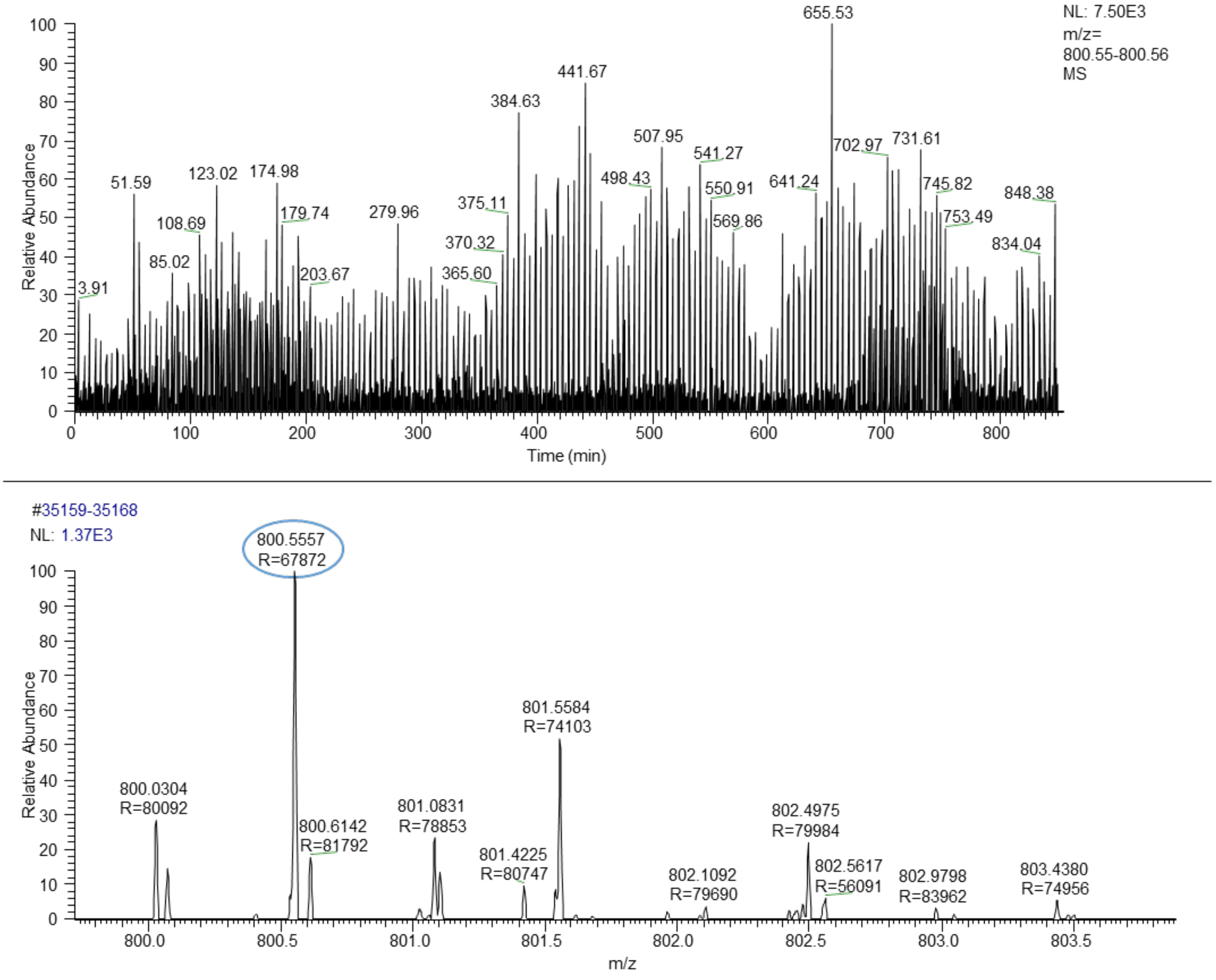

Figure S4: Exemplary MALDI spectrum. In the upper part, the extracted ion chromatogram of m/z 800.5557 is shown. The lower part shows the summed spectrum. The ion of interest is marked with a blue circle. According to the LC-MS/MS data, this peak belongs to PC(16:0_18:0) $[M+K]^+$.

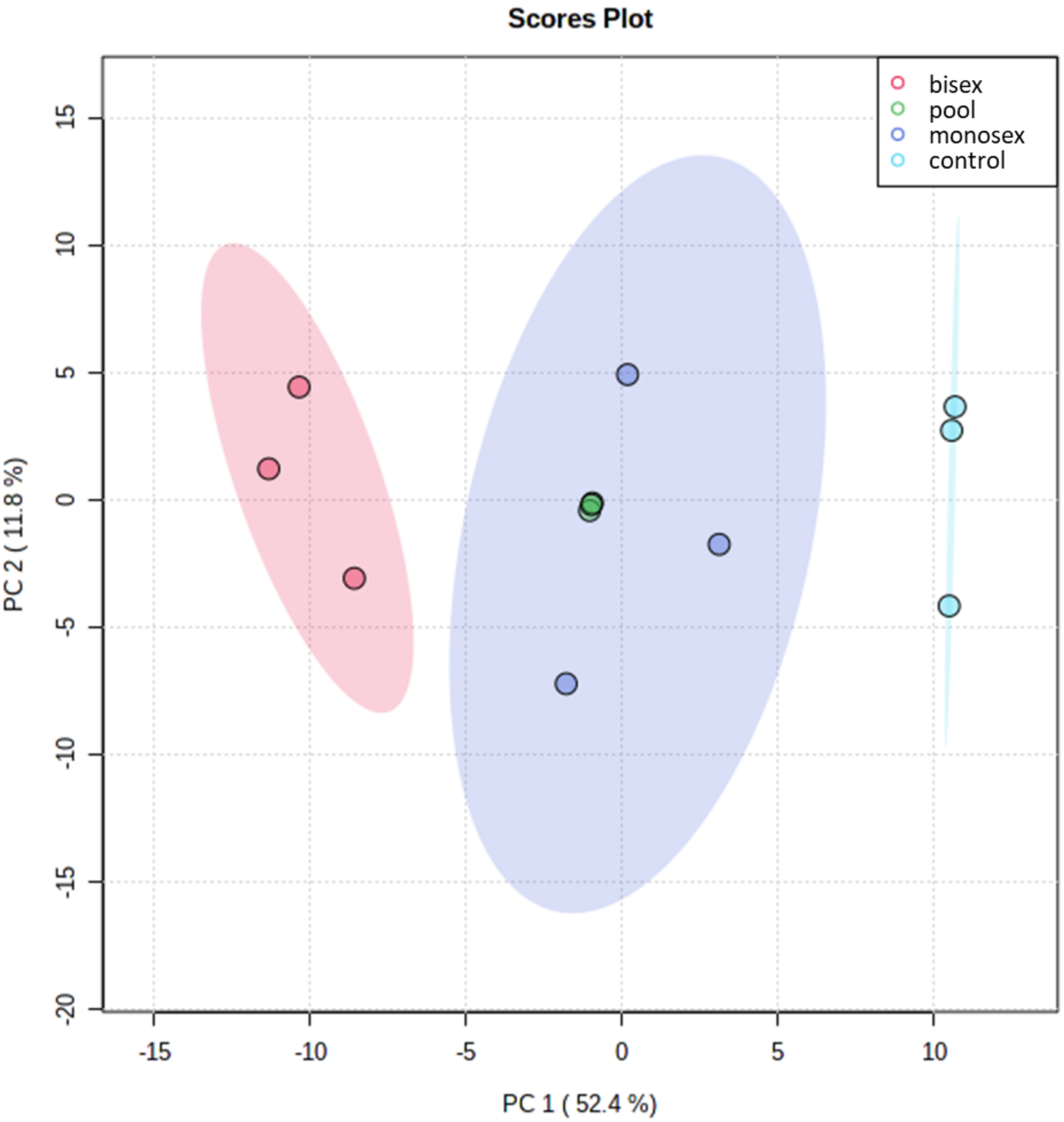

Figure S5: PCA scores plot from MetaboAnalyst[36]*, showing that the three sample groups are clearly distinguishable. The pool samples group nicely on nearly the same point, what is reasonable since they are only technical replicates.*

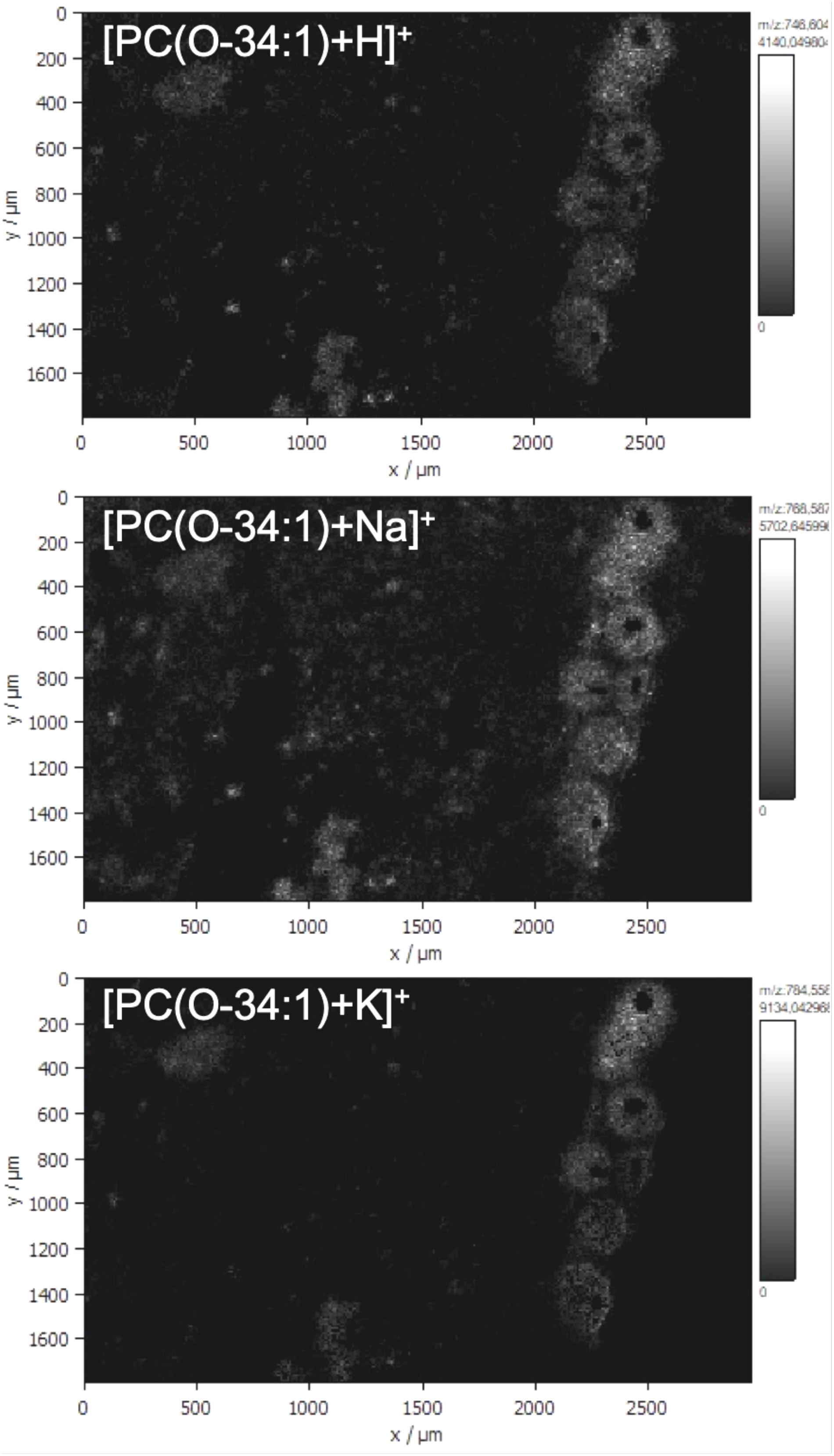

Figure S6: Comparison of different adducts for one lipid. Nearly the same distribution patterns were found. However, for most of the lipids, not all adducts were observed.

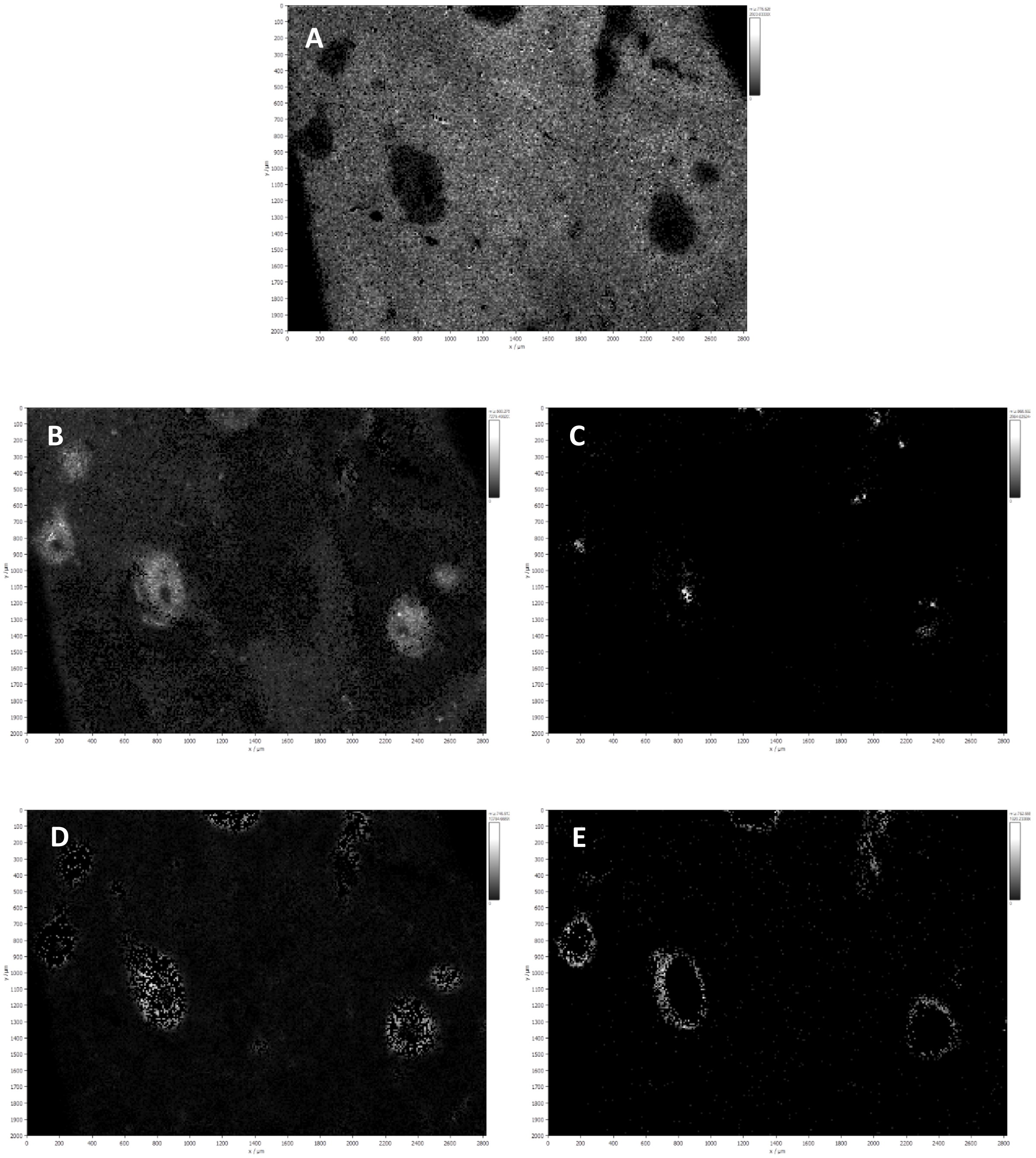

Figure S7: Single-ion images that were used for overlays of a bisex-infected sample as shown in Figure 5. A: m/z 776.526946, MMPE(16:0_22:6), [M−H]⁻, B: m/z 500.275684, LPE(20:4), [M−H]⁻, C: m/z 866.592639, PS(42:4), [M−H]⁻, D: m/z 746.511353, plasmenyl-PE(P-16:0/22:6), [M−H]⁻, E: m/z 752.555581, plasmanyl-PE(O-18:0/20:4), [M−H]⁻.

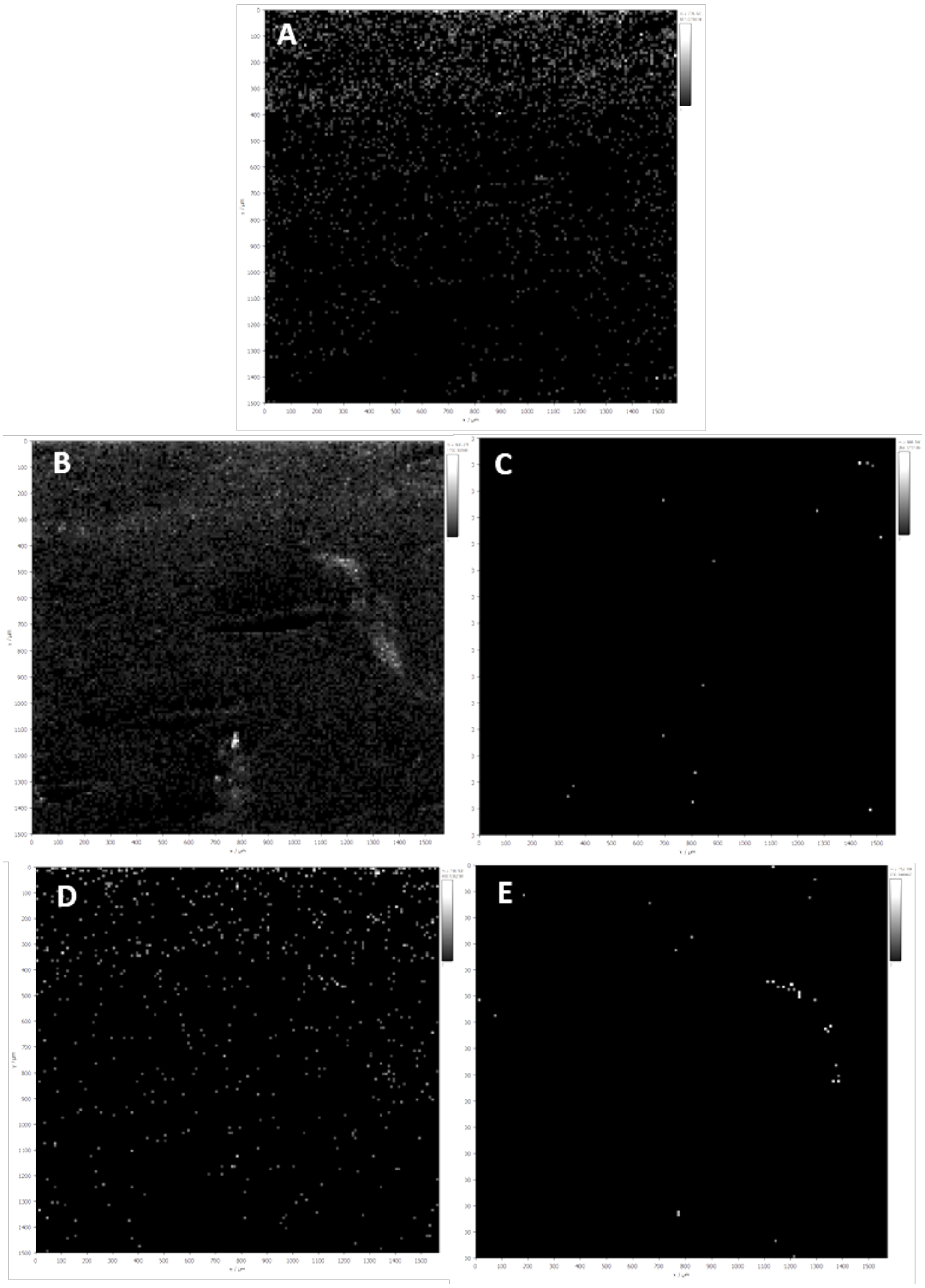

Figure S8: Single-ion images that were used for overlays of a control sample as shown in Figure 5. A: m/z 776.526946, MMPE(16:0_22:6), [M–H]⁻ , B: m/z 500.275684, LPE(20:4), [M–H]⁻ , C: m/z 866.592639, PS(42:4), [M−H]⁻, D: m/z 746.511353, plasmenyl-PE(P-16:0/22:6), [M–H]⁻ , E: m/z 752.555581, plasmanyl-PE(O-18:0/20:4), [M–H]⁻.

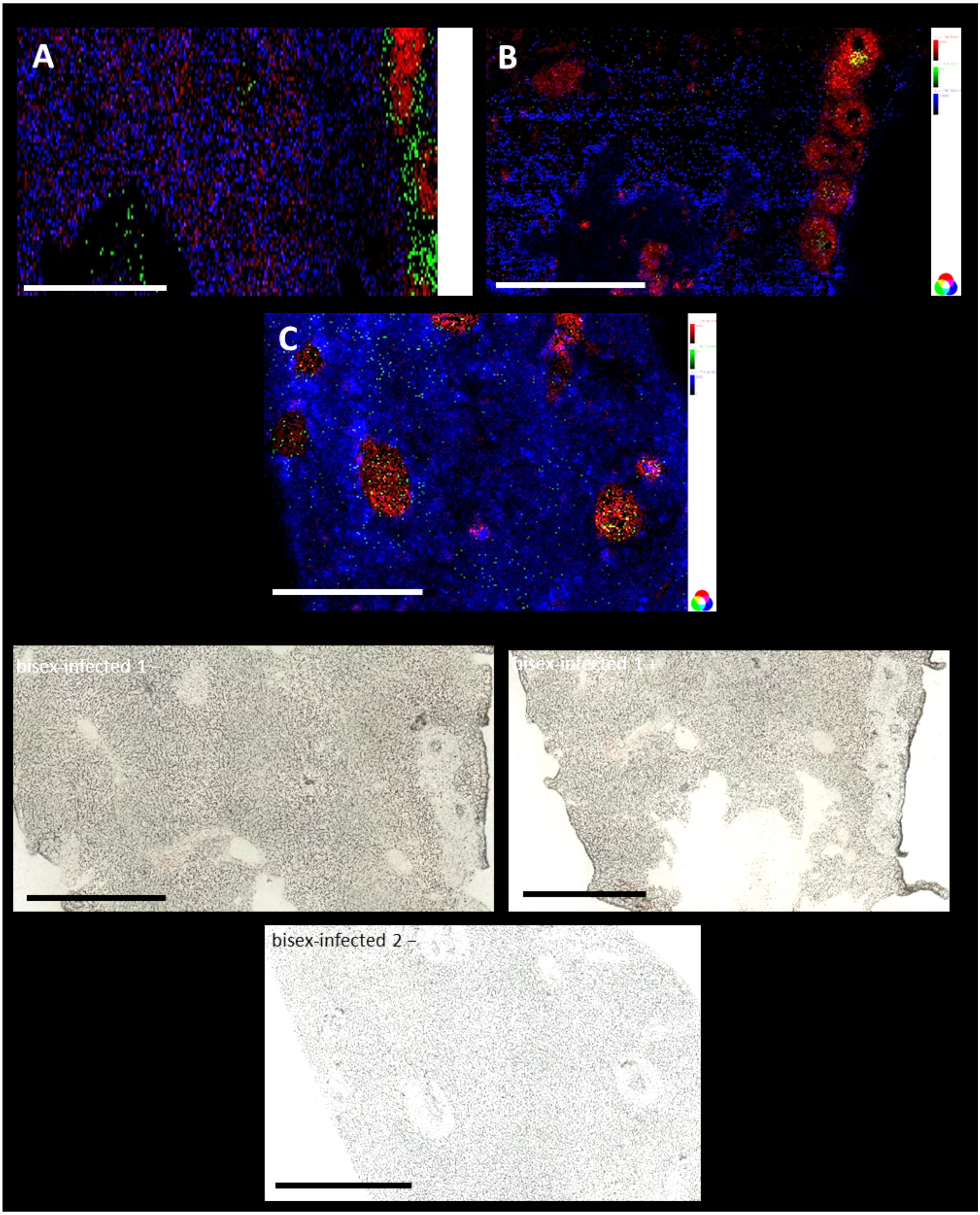

Figure S9: Upper part: Overlay MS images based on markers found by LC-MS/MS analysis. For A, m/z 836.545472, tentatively assigned to PS(40:5), [M–H]$^-$ was found to be a marker for granuloma, m/z 788.529391, DMPE(18:3_20:4), [M–H]$^-$ a marker for surrounding tissue and m/z 752.557091, plasmanyl-PE (O-18:0_20:4), [M–H]$^-$ a marker for the outer part of the granuloma. For B, m/z 746.604414, plasmanyl-PC(O-16:1_18:0), [M+H]$^+$ was found to be a marker for granuloma, m/z 780.550319, PE(17:0_22:5), [M+H]$^+$ a marker for surrounding tissue and m/z 438.297017, plasmenyl-LPE (P-16:0), [M+H]$^+$ as a marker for an enrichment inside the granuloma. For C, m/z 728.561836, plasmenyl-PE(P-18:0_18:1) or plasmanyl-PE(O-18:0_18:2), [M–H]$^-$ as a marker for granuloma and m/z 867.520849, PG(22:5_22:6), [M–H]$^-$ as a marker for an enrichment inside the granuloma. m/z 774.541653, plasmenyl-PE(P-18:0_22:6), [M–H]$^-$ was taken as a marker for surrounding tissue. Lower part: Corresponding microscopically images. Scale bars are 1 mm.

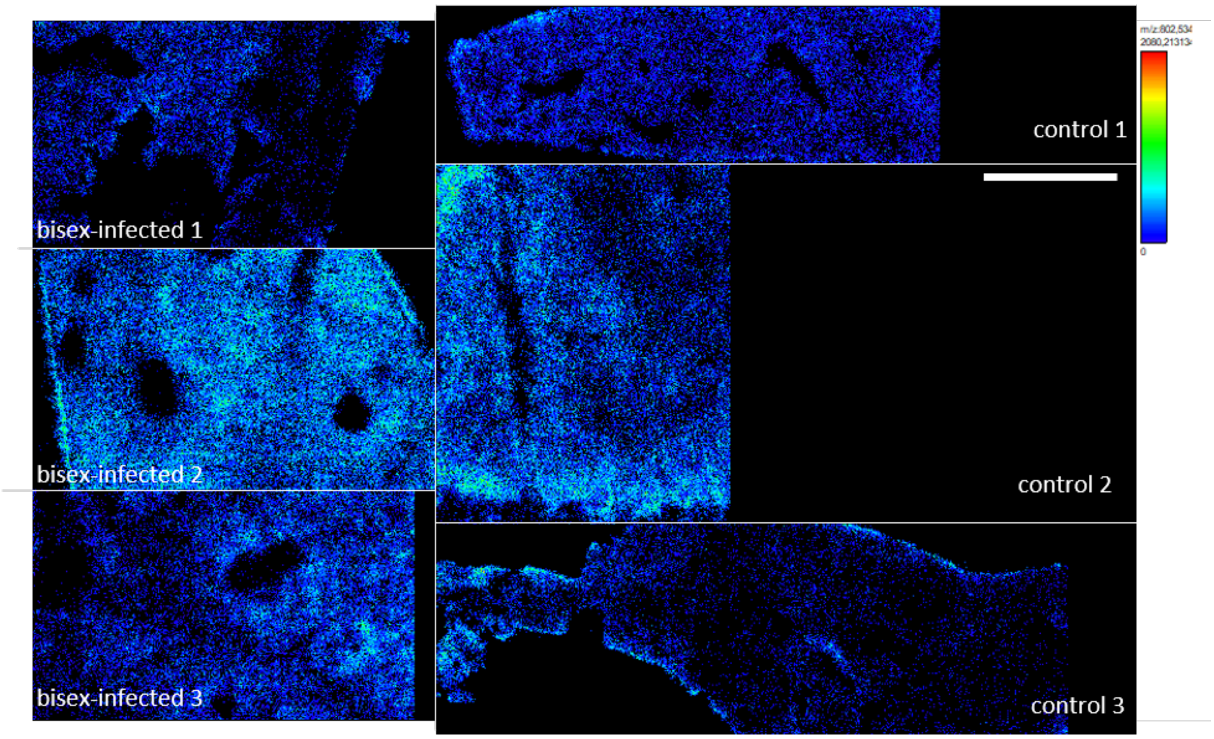

Figure S10: Lateral distribution of PC(16:0_20:5) at m/z 802.535727 as [M+Na]+. While the lipid is depleted in the area of granulomas of liver samples of bisex-infected hamsters (left), it is evenly distributed in the control samples (right). Scale bars are 1 mm.

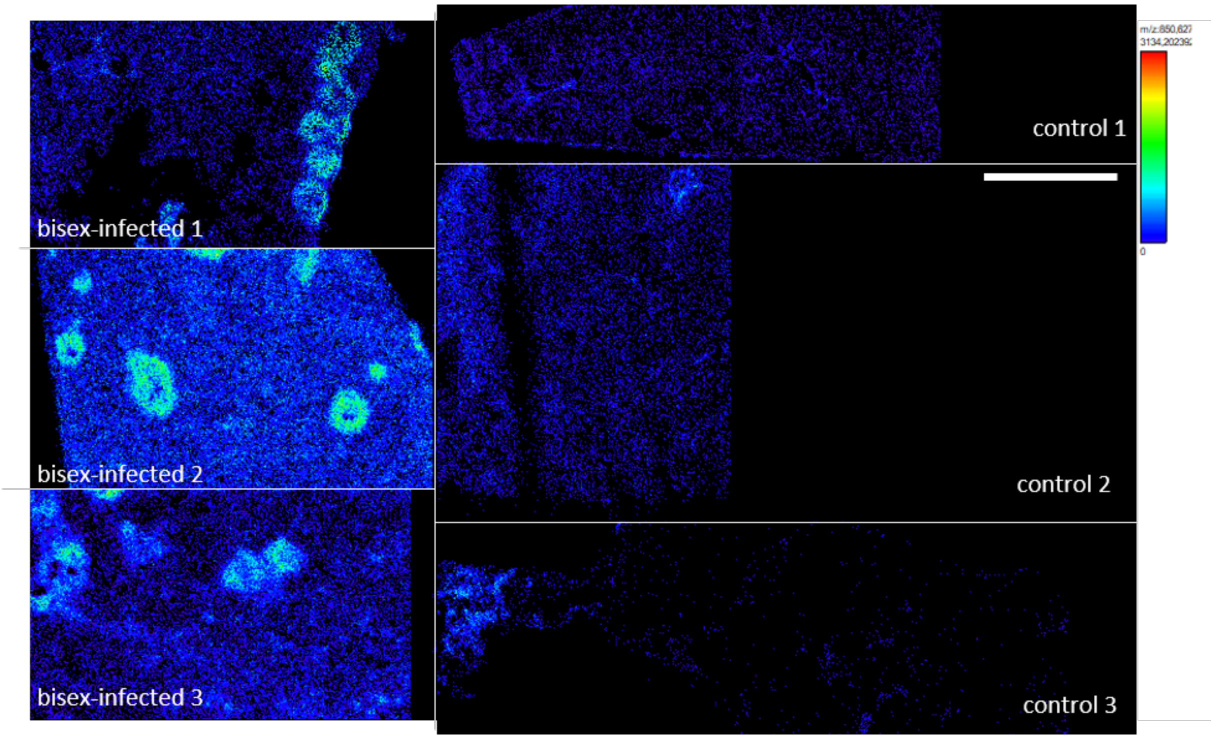

Figure S11: Lateral distribution of PC(19:0_20:2) at m/z 850.629627 as [M+Na]+. While the lipid is evenly distributed in the control samples (right), enrichment in the granulomas is recognizable in liver samples of bisex-infected hamsters (left). However, the lipid is still detectable in the non-affected parts of the tissue. Scale bars are 1 mm.

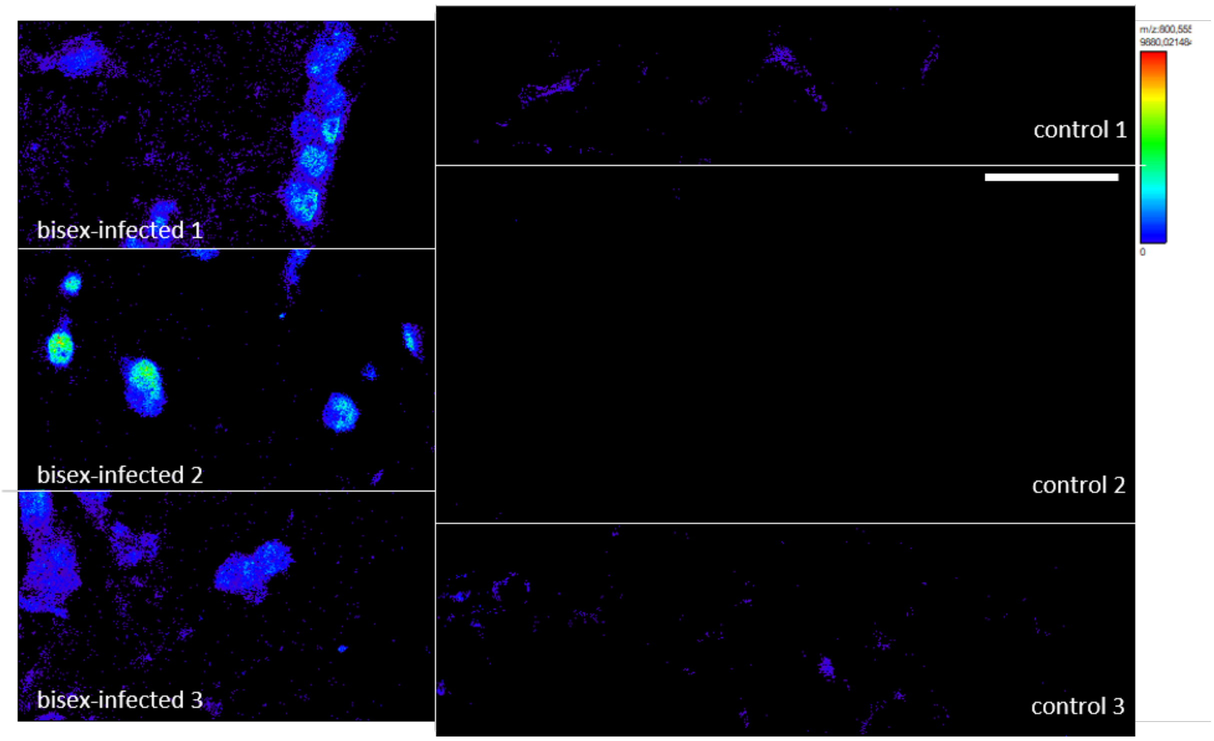

Figure S12: Lateral distribution of PC(16:0_18:0) at m/z 800.556617 as [M+K]+. While the lipid was not found in the control samples (right), strong enrichment in the granulomas is recognizable in liver samples of bisex-infected hamsters (left). The lipid species was found accumulated especially in direct contact around the eggs. Scale bars are 1 mm.

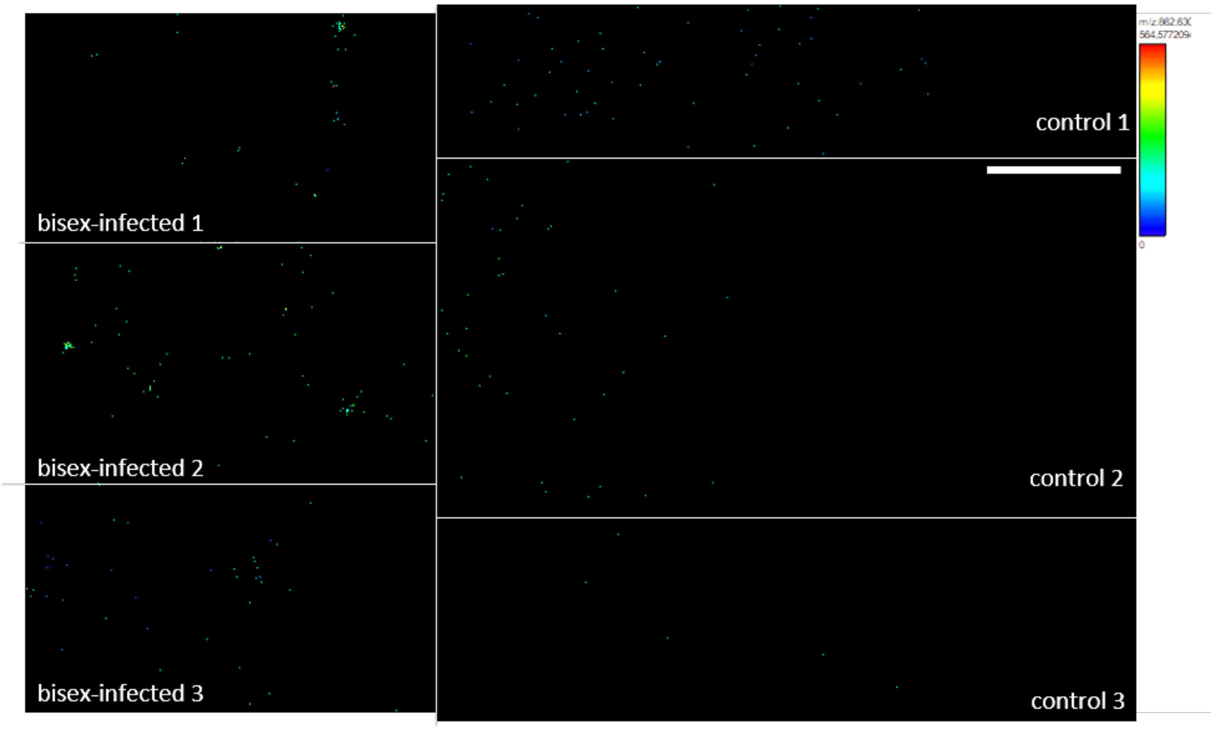

Figure S13: Lateral distribution of PC(20:0_20:3) at m/z 862.630305 as [M+Na]+. The lipid was neither found in the control samples, nor in the non-affected tissue of liver samples of bisex-infected hamsters but was only found in and directly around the eggs. Scale bars are 1 mm.

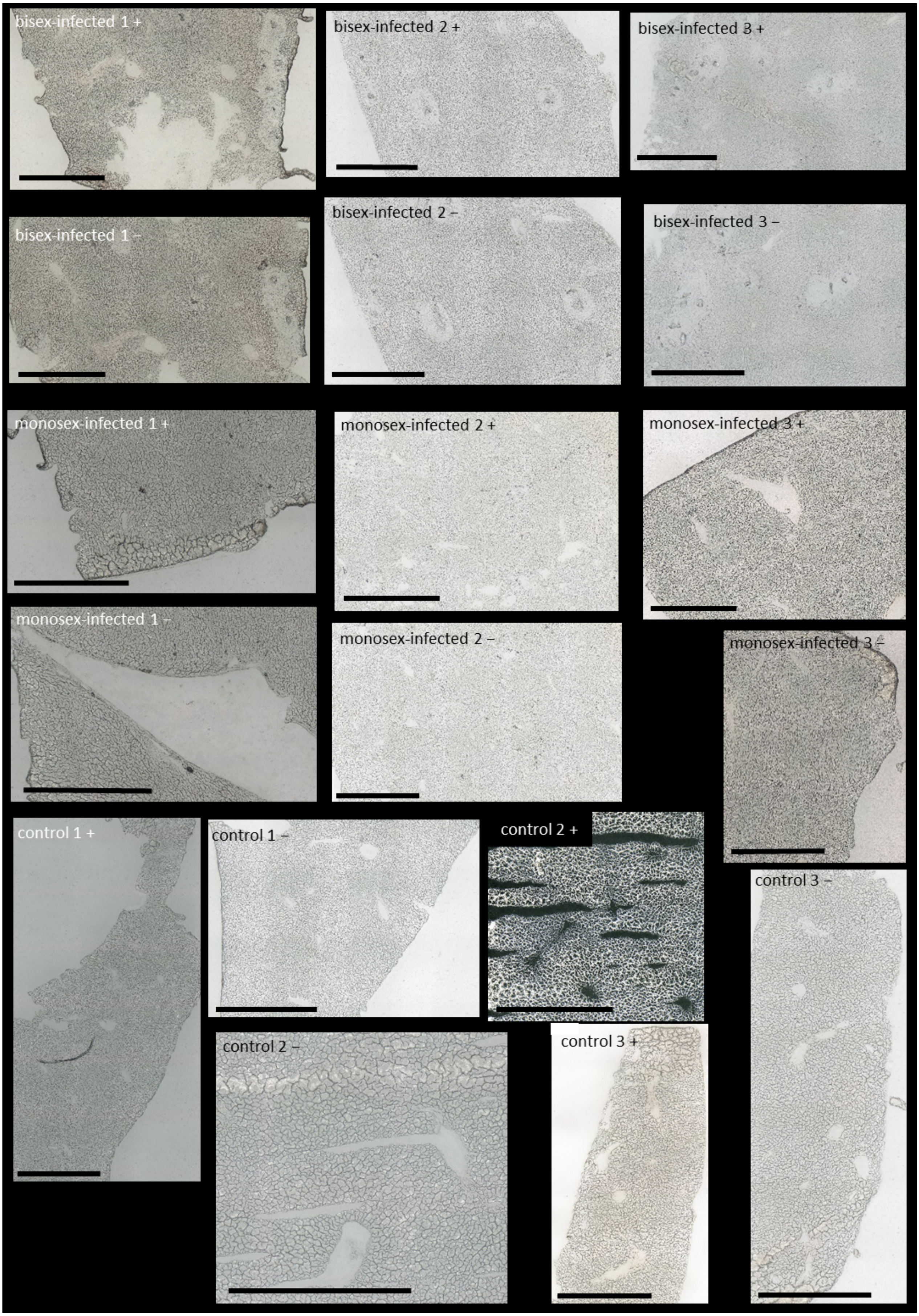

Figure S14: Microscopical images of all samples used for MALDI MSI experiments. Pictures were taken before matrix application and measurement. Scale bars are 1 mm.
